# Extended fractional Hofstadter states at high field in twisted bilayer graphene above the magic angle

**DOI:** 10.1073/pnas.2602080123

**Published:** 2026-10-05

**Authors:** Joe Finney, Aaron Sharpe, Linsey K. Rodenbach, Jian Kang, Xiaoyu Wang, Kenji Watanabe, Takashi Taniguchi, Marc A. Kastner, Oskar Vafek, David Goldhaber-Gordon

**Affiliations:** ^a^ Department of Physics, Stanford University, Stanford, CA 94305; ^b^ Stanford Institute for Materials and Energy Sciences, SLAC National Accelerator Laboratory, Menlo Park, CA 94025; ^c^ School of Physical Science and Technology, ShanghaiTech University, Shanghai 200031, China; ^d^ National High Magnetic Field Laboratory, Tallahassee, FL 32310; ^e^ Research Center for Functional Materials, National Institute for Materials Science, Tsukuba 305-0044, Japan; ^f^ International Center for Materials Nanoarchitectonics, National Institute for Materials Science, Tsukuba 305-0044, Japan; ^g^ Department of Physics, Massachusetts Institute of Technology, Cambridge, MA 02139; ^h^ Department of Physics, Florida State University, Tallahassee, FL 32306

**Keywords:** twisted bilayer graphene, fractional Chern insulator, Hofstadter’s butterfly

## Abstract

When a Chern band is partially filled, a fractional Chern insulator (FCI)—the lattice analog of a fractional quantum Hall state—can arise. Though the full range of possible scenarios for producing such a state is not established, the most tractable models combine strong electronic interactions with the quantum geometry of the parent Chern band meeting specific criteria. In twisted bilayer graphene, the importance of interactions can be tuned by varying the interlayer twist. Here, we study a sample with twist near 1.4^°^, large enough to suppress the zero-field correlated states. We find that applying a strong magnetic field restores the importance of electron–electron interactions: at nearly half a magnetic flux quantum per moiré unit cell, deep in the Hofstadter regime, odd-denominator fractional states appear in multiple Hofstadter subbands. These fractional states persist over larger ranges of density, and are more robustly quantized, than nearby integer states, opposite to what is seen in other fractional quantum Hall and FCI systems.

## Significance

Electrons in solids can collectively organize into topological states characterized by integer or fractionally quantized Hall conductance. Fractional states, which rely on strong electron–electron interactions, are typically more fragile than their integer counterparts. Here, we report the finding of fractional states in twisted bilayer graphene, two layers of graphene that are slightly rotated. At extremely high magnetic fields we find a surprising reversal of the usual hierarchy: numerous fractional states are more robustly quantized than nearby integer states. Particular fractional states persist across an unusually wide range of charge density, pointing to an unconventional stabilization mechanism that may involve charge ordering. These findings identify a new regime in which fractional topological phases can be robust.

Under the right conditions, the Bloch bands formed from electronic wavefunctions in a periodic potential can acquire nontrivial topology. These “Chern” bands are analogous to Landau levels on which quantum Hall states are based, but do not require a magnetic field. If interactions are strong, a partially filled Chern band can host a fractional Chern insulator (FCI),^*^ the lattice analog of a fractional quantum Hall (FQH) state (1–8). Only a few examples of materials hosting FCIs have been experimentally reported: twisted MoTe_2_ (9–11) and graphene-based multilayers aligned with hexagonal boron nitride (hBN) (12–16). The graphene-based FCIs appear very sensitive to structural parameters; it is not known why particular configurations exhibit FCIs whereas others do not despite having flat bands with nontrivial quantum geometry (17, 18).

Twisted bilayer graphene (TBG), the archetypal strongly correlated moiré heterostructure, displays astonishingly diverse correlated electronic and topological phases (13, 19–29). Whether these can include FCIs remains an open question (30). The electronic moiré miniband structure of TBG depends sensitively and predictably on twist angle (31). Near the magic angle of 1.1^°^, the large ∼10 nm moiré length scale enables tuning the density of charge carriers through entire minibands and the density of magnetic flux to on order one flux quantum $\Phi_{0}=h/e$ per moiré cell, where *e* is the elementary charge and *h* is Planck’s constant. In this “Hofstadter’s butterfly” regime (12, 25, 28, 29, 31–42) the energy spectrum exhibits a fractal structure with field-induced topological subbands. Gaps in the fractal spectrum appear at fluxes and densities described by Diophantine equations of the form $n/n_{s}=s+t\Phi /\Phi_{0}$ (43). Here, $n_{s}=1/A$, Φ=BA, *A* is the moiré unit cell area, *B* the magnetic field normal to the plane of the samples, *s* the density offset at zero flux, and *t* the Chern number associated with the gap. We notate these Středa lines as (s, t). Within such a gap, the Hall conductance is expected to be quantized to $\sigma_{\mathrm{xy}}=te^{2}/h$ (44).

Fractional Hofstadter states (FHSs) are associated with Středa lines whose *t* is fractional, and whose *s* does not coincide with the charge neutrality point or a band edge (2–8, 45). Because Hofstadter subbands generically carry nonzero Chern numbers, an FHS is the finite-field counterpart of an FCI. Recently, fractional *t* states have been observed in high-quality magic-angle TBG (46). These occurred in Landau levels originating from charge neutrality (s=0), just like conventional FQH. In TBG, FHSs with s≠0, reflecting an underlying broken symmetry, have only been clearly demonstrated in samples in which the graphene lattice is aligned with that of hBN (12, 13), a known source of symmetry breaking for electrons in graphene.

In refs. 47 and 48, we presented magnetotransport measurements and single-particle calculations for a TBG sample twisted to near 1.37^°^, where the miniband width is large enough (near 100 meV) to suppress electronic correlations near zero magnetic field (19, 21, 47).

In this work, we further explore that same sample, now at extremely high magnetic fields, demonstrating many *B*-induced strongly correlated states. Most notably, at magnetic flux near 2/5 a magnetic flux quantum per moiré unit cell we observe plateaus in Hall resistance corresponding to t=±8/3 and −8/5, quantized to within a few tenths of a percent, along with possible −4/3 plateaus. The ±8/3 plateaus in particular extend over a strikingly broad density range, up to half an electron per moiré unit cell, rather than following a narrow Středa line. Since the early days of quantum Hall, in some samples integer quantum Hall states have extended over a broad density range, outcompeting fractional states that might be expected at those densities. This “reentrant quantum Hall effect” has been explained as a consequence of a Wigner crystal coexisting with a filled Landau level (15, 49, 50), a scenario originally termed a partial Hall crystal (51). In our present work, in contrast with prior reports, the integer states are *less* robust and broad than the 8/3 fractional states. One possible explanation for the surprising extended FHS region is the formation of a fractional partial Hall crystal.

## 1. High-Field Magnetotransport

**Fig. 1.**
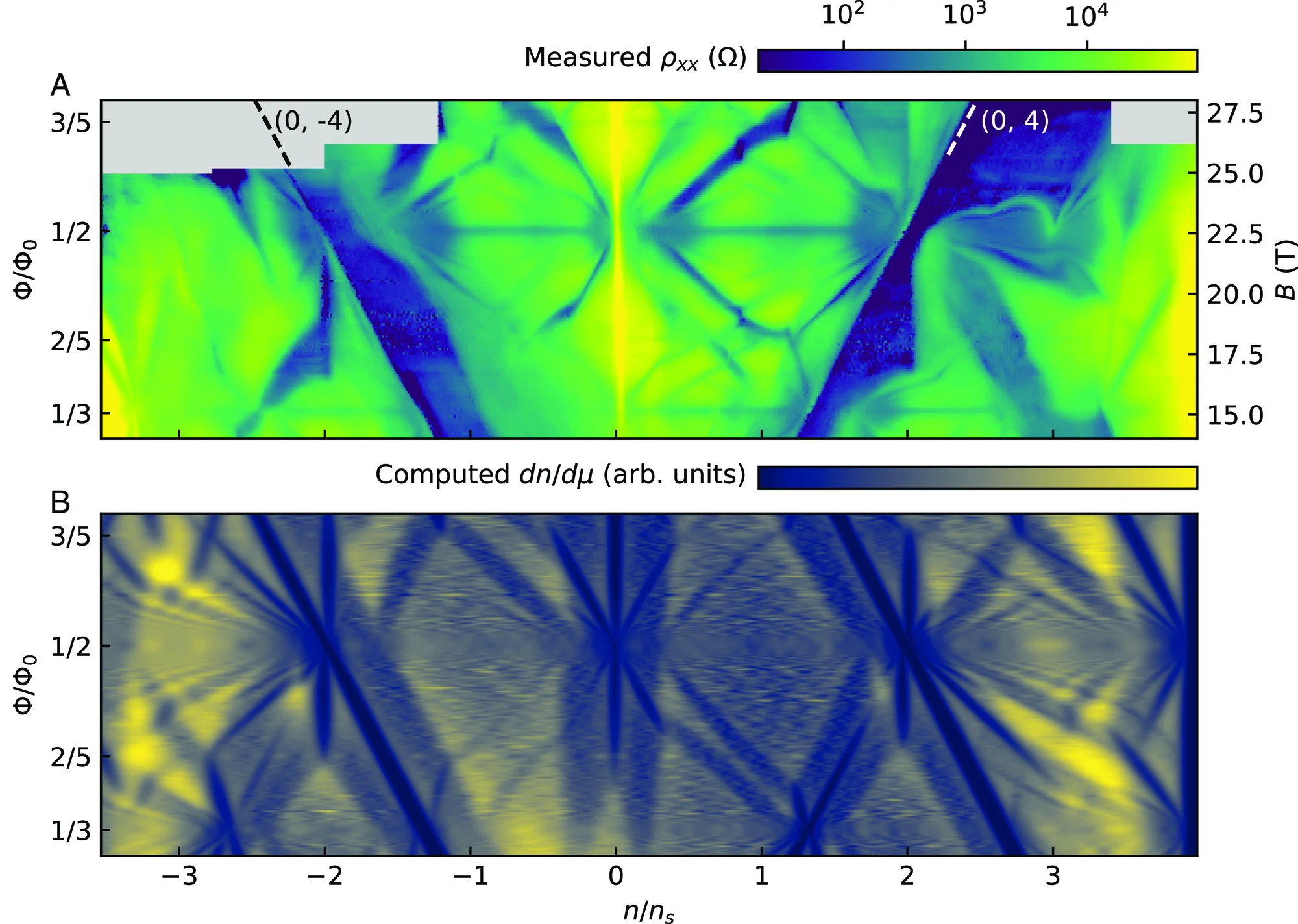
Strained TBG in the Hofstadter butterfly regime. (*A*) Dependence of the longitudinal resistivity as a function of density and field. We indicate Středa lines (s, t)=(0,±4) with dashed lines. The metallic top gate began to leak to the TBG above 25 T, so we reduced its voltage range, leaving the grayed-out regions. See the Supplement for a schematic description of quantum Hall gaps (*SI Appendix*, Fig. S4), the behavior of other contact pairs (*SI Appendix*, Figs. S5 and S6), a comparison with the lower-field data presented in ref. 47 (*SI Appendix*, Fig. S3), and a schematic of the device with our inferred twist angles as extracted from the Landau fan diagram (*SI Appendix*, Fig. S2). (*B*) Computed density of states d*n*/d*μ* for the spectrum in Fig. 3*A*. Gaps show up as dark lines. See Section 3 for details on the computation.

In our sample, longitudinal resistivity as a function of carrier density and of magnetic field between 14 and 28 T ranges from below our measurement floor of a few ohms within quantum Hall gaps to hundreds of kilohms at band edges and near charge neutrality (Fig. 1*A*). Here, we focus on specific contact pairs within this 20-terminal Hall bar that demonstrated the sharpest low-field transport in ref. 47. For our twist angle of 1.37^°^, $\Phi /\Phi_{0}=1$ occurs at nearly 45 T, so the field range in our high-field measurements corresponds to flux ratios between 0.31 and 0.62. We indicate the most prominent Středa lines, (s, t)=(0,±4), which persist without closing down to near-zero field (*SI Appendix*, Fig. S3).

For comparison, we show the computed Wannier plot in the same field range (Fig. 1*B*). The computed density of states dn/dμ should show patterns similar to those seen in resistivity, but we do not directly calculate transport from our model as we did in ref. 48 for lower magnetic fields. Our model, which incorporates uniaxial heterostrain, Zeeman effect, and electron–hole asymmetry, broadly matches our experimental observations of splitting and bending behavior of Landau levels emanating from half flux and $n/n_{s}=0$. The model predicts several prominent gaps that we discuss in Section 3 together with associated experimental measurements.

In the Supplement we describe other striking phenomena such as a Landau level reset at half flux, Brown-Zak oscillations, symmetry-broken Chern insulating Středa lines with (s,t)=(±1/2,±3), and correlated Hofstadter ferromagnets.

## 2. Fractional Hofstadter States

**Fig. 2.**
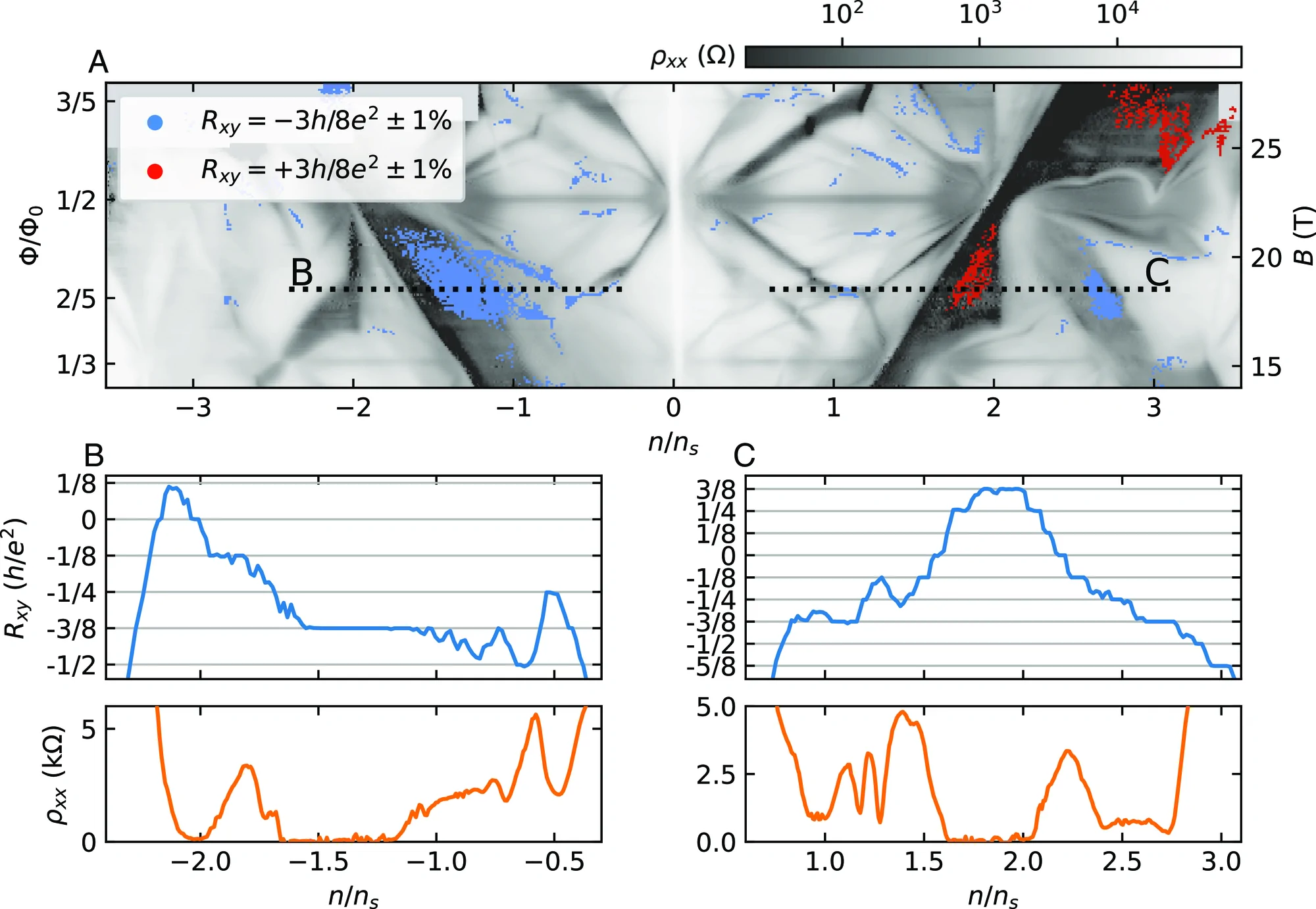
Hall plateaus of fractional Hofstadter states. (*A*) Longitudinal resistivity from Fig. 1 replotted in gray-scale. Colored regions superimposed on the gray-scale plot mark where the Hall resistance for a neighboring contact pair is quantized to within 1% of −8/3 (blue) or +8/3 (red). A clustering algorithm has been applied to remove spurious points (*SI Appendix*, section S9). The abrupt loss of quantization at $\Phi /\Phi_{0}$ between 1/3 and 2/5 corresponds to a pause in the measurement of several hours. This, along with the noise above 1/2, are described in *SI Appendix*, section S1. (*B* and *C*) Hall resistance and longitudinal resistivity as a function of density at 18.5 T ($\Phi /\Phi_{0}=0.41$, black dashed lines in *A*).

In Fig. 2*A*, we overlay in blue and red the regions where the Hall resistance is quantized to within 1% of $-3\;h/8e^{2}$ and $+3\;h/8e^{2}$ respectively. We apply an intentionally conservative clustering threshold algorithm so that almost all of the pockets marked with color represent well-quantized plateaus of ±8/3 rather than regions where the Hall resistance incidentally passes through quantized values. These fractional Hall plateaus coincide with low longitudinal resistivity (shown in gray). See *SI Appendix*, section S9 for details and *SI Appendix*, Fig. S17 for unfiltered data.

Fig. 2, panels *B* and *C* show line cuts of $R_{\mathrm{xy}}$ and $\rho_{\mathrm{xx}}$ at 18.5 T. The aforementioned ±8/3 plateaus are most prominent, but integer plateaus are visible, along with a small −8/5 plateau. For a discussion of the degree of quantization, other fractions, and the behavior in other contact pairs, see *SI Appendix*, sections S6, S10, and S11, respectively.

We find that regions where the Hall resistance is (fractionally) quantized typically coincide with suppressed longitudinal resistivity in the four adjacent contact pairs (Fig. 1 and *SI Appendix*, section S6). Though measurements from neighboring longitudinal probes vary subtly, neighboring Hall probes exhibit strikingly different behavior: those just 3 microns away from the main Hall pair we focus on in this manuscript exhibit poor quantization, even for integer quantum Hall states (*SI Appendix*, Fig. S14), possibly due to mixing of the longitudinal resistivity into the Hall measurement because of spatial variation in twist angle.^†^ We therefore suspect that the main Hall pair of this manuscript contacts a region of unusually high moiré uniformity.

We defer a detailed discussion of potential explanations of the extended fractional phenomenology to Section 4.

## 3. Hofstadter Model

To understand the Hofstadter bands we would expect without interactions we perform bandstructure computations within a single-particle model. Our effective continuum model from ref. 48, which accounted for uniaxial heterostrain, can be extended into the Hofstadter regime (41). Here we also incorporate biaxial heterostrain, lattice relaxation, and electron–hole asymmetry so that the locations of the three van Hove points near B=0 on the hole side of the charge neutrality point differ from those on the electron side (52–54), as is seen in the experiment (47, 48). We fully parameterize the 2×2 moiré deformation matrix as a function of twist angle, uniaxial heterostrain magnitude and direction, and biaxial heterostrain magnitude. We then search for structural parameters that yield bands with van Hove points at densities matching those identified at zero field in ref. 48. We find a unique best fit—0.24±0.02% uniaxial heterostrain at 45±4^°^ and 0.3±0.1% biaxial heterostrain—that places all six computed van Hove densities within our experimental bounds. See *SI Appendix*, section S13 for details.

**Fig. 3.**
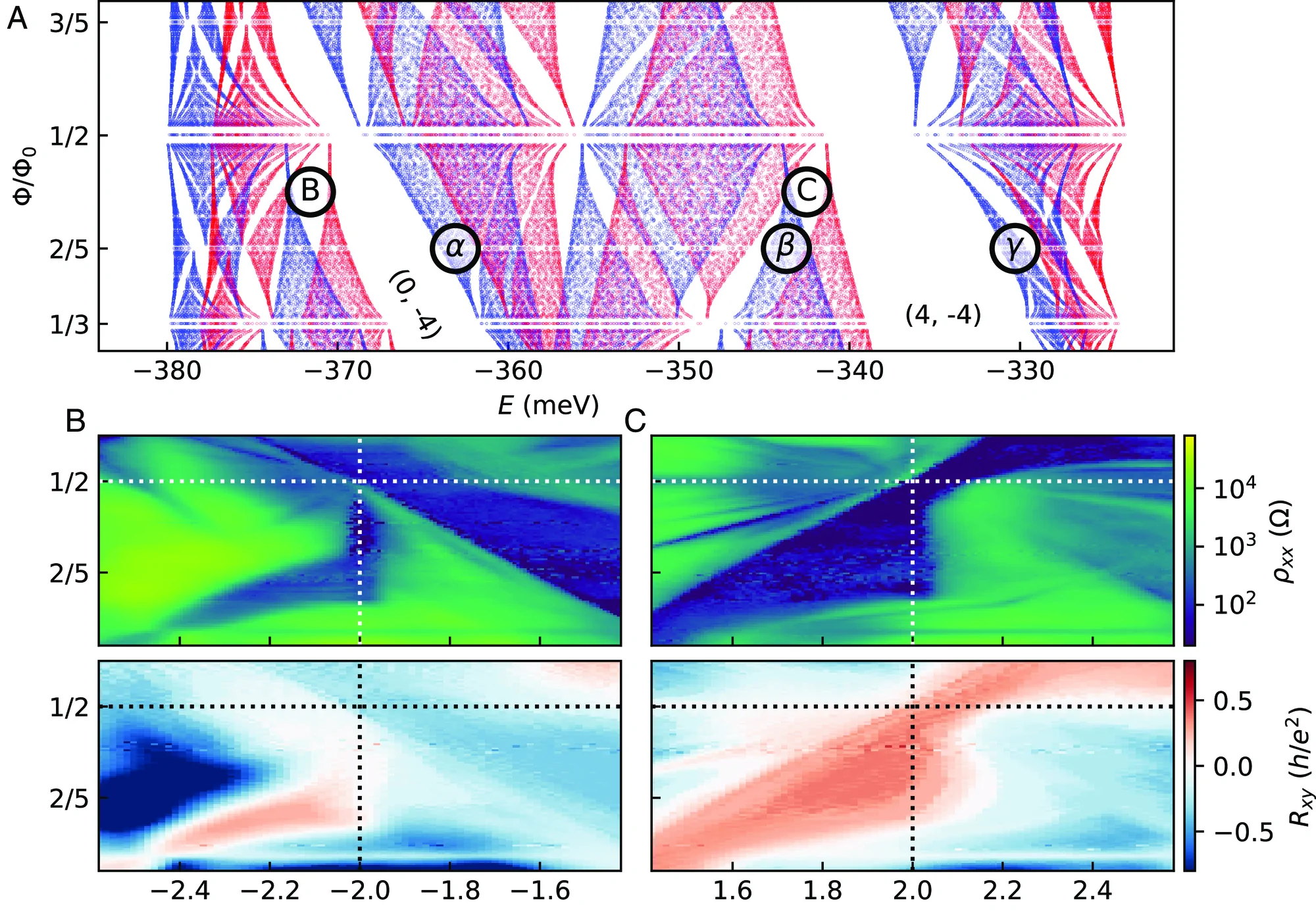
Computed Hofstadter spectrum with gaps near half flux, at fixed carrier density. (*A*) Computed energy levels for K valley of strained TBG (q≤72), as described in the text. Parameters: $\theta =1.35^{{}^{\circ}}$, 0.24% uniaxial heterostrain at 45^°^ and 0.3% biaxial heterostrain. Spin up electrons are shown in blue, and spin down electrons are shown in red. The gaps at (s,t)=(0,−4) and (4,−4) are labeled. *α*, *β*, and *γ* denote the Hofstadter subbands which host the largest pockets of ±8/3 quantization shown in Fig. 2. Two gaps (s,t)=(∓2,0) are denoted B and C, respectively. See *SI Appendix*, Fig. S22 for the full range of fields. (*B* and *C*) Dependence of the longitudinal resistivity (*Top* panels) and Hall resistance (*Bottom* panels) as a function of density and field centered on the two gaps predicted in (*A*). In (*B*), below half flux we observe a strong suppression of longitudinal resistivity at fixed density $n/n_{s}=-2$ over a range of flux, coincident with a small plateau of zero Hall resistance. In (*C*), we observe low longitudinal resistivity along a vertical line. However the Hall resistance is not zero at the same densities.

Fig. 3*A* shows the Hofstadter spectrum computed for this set of moiré parameters, over a range of flux filling corresponding to the magnetic field range of our measurements. We include a Zeeman splitting term $\Delta E=g\mu_{B}B$, with g=2 as expected for electron spins in graphene or other forms of carbon (55). Spin-up electronic states (aligned with the external field) are shown in blue, and spin-down states in red. Computing the density of states d*n*/d*μ* of this spectrum facilitates comparison with our transport data (Fig. 1): the computed density of states qualitatively captures the extent in field and density of many well-formed gaps and subtler features in transport. However, not every feature in our noninteracting theory calculations matches experiment. Notably, the calculations show (4, −4) and (0, −4) as more prominent gaps than (0, 4) and (−4, 4). In transport the (0, −4) Středa feature is indeed more prominent than (−4, 4). But the (0, 4) Středa feature is more prominent than (4, −4) (Fig. 1*A*), suggesting band renormalization effects beyond the single-particle picture (56).

Labels (B) and (C) in Fig. 3*A* denote two gapped states predicted by our model. Each extends over a range of magnetic fields near half flux, at fixed finite filling (∓2,0), respectively. In each case, just below half flux the Zeeman splitting exceeds the bandwidth of a subband separating two gaps associated with Středa lines that cross at half flux. The Středa lines that cross at (B) are parameterized by (−4, 4) and (0, −4), and those at (C) by (0, 4) and (4, −4). In transport, we observe suppression of $\rho_{\mathrm{xx}}$ in a narrow vertical strip at $n/n_{s}=\mp 2$, emanating downward from half flux over a magnetic field range similar to that predicted by our model (Fig. 3 *B* and *C*, *Upper* panels). $\rho_{\mathrm{xx}}$ often reaches our measurement floor of 1 Ω (in other contact pairs we see similar features but the minima are higher, see *SI Appendix*, Fig. S5). At (B), the Hall resistance is zero below half flux near $n/n_{s}=-2$ (Fig. 3*B*, *Lower* panel), consistent with the vanishing Hall conductance expected for a t=0 gap. At (C), however, the Hall resistance is not zero near $n/n_{s}=2$ (Fig. 3*C*, *Lower* panel). We see similar vertical features in $\rho_{\mathrm{xx}}$ at quarter flux where the (±8, ∓8) and (0, ±8) gaps intersect (*SI Appendix*, Fig. S3).

## 4. Discussion

We turn now to the 8/3 fractional state which surprisingly persists over a larger range of density than any integer state. A linecut at 18.5 T, near 2/5 flux (Fig. 2 *B* and *C*), traverses the three largest pockets of quantization in the Landau fan diagram, centered at $n/n_{s}=-1.3,1.9$, and 2.6. These occur upon doping into the next band above the Hofstadter gaps (0,−4), (0,+4), and (+4,−4), respectively. Assuming the total Chern number ±8/3 extracted from measured Hall resistance is the sum of the Chern number Δt of the FHS and that of the gapped integer state below, the Chern numbers of the three FHSs would be +4/3=−8/3−(−4), −4/3=8/3−4, and again +4/3=−8/3−(−4), respectively. In Fig. 3*A*, within our single-particle model we denote the parent spin-polarized Hofstadter subbands at 2/5 flux that we believe host the FHSs *α*, *β*, and *γ*, respectively. If interactions do not generate substantial mixing of bands, in each case we are doping into a spin-polarized (all blue in the figure) but valley-degenerate band. In this scenario, the experimentally extracted 4/3 Chern number represents two copies of a |Δt|=2/3 FHS. Alternatively, if valley degeneracy is spontaneously broken by interactions the observed quantization could reflect a nondegenerate |Δt|=4/3 FHS. We cannot rule out the possibility that it is four copies of a 1/3 FHS, but that would require recovery of spin degeneracy in the face of a Zeeman energy comparable to subband widths.

Unlike ordinary Landau levels, the three subbands we postulate as parents for the FHSs have finite bandwidth, even without disorder. Subband *γ* is the narrowest at ∼1 meV. The others have width ∼2 meV at 2/5 flux, and the associated FHSs extend over a range of flux for which the bands broaden further and even overlap with nearby subbands, factors we would ordinarily expect to disfavor formation of FHSs.

Subband *α* and its corresponding Hall plateau are particularly enigmatic. We observe an enormous region of quantization ($\Delta n/n_{s}∼0.5$), larger than any other plateau—integer or fractional—between 0 and 28 T (*SI Appendix*, Fig. S16). An incompressible gap is expected to follow a well-defined Středa relation with slope equal to the total Chern number of the occupied bands. However, the breadth of this plateau stymies assigning a specific (*s*, *t*). To retain quantization as carrier density is tuned, the excess carriers must go into localized states. What states are we doping into here? The obvious culprit would be localized states from disorder. Though we cannot explicitly rule out this scenario, it is unlikely that localized states from disorder would specifically extend these ±8/3 fractional gaps so much more dramatically than other gaps in the spectrum. In *SI Appendix*, sections S12 and S14, we discuss the effects of domain walls and spin-dependent transport, respectively. We consider both scenarios possible but unlikely as explanations of our data.

A more likely reason for these fractional plateaus to be so large is the formation of a fractional partial Hall crystal: a Wigner crystal in a FHS background. In this scenario, upon doping with respect to primitive filling of the FHS gap, the strongly interacting dilute system of quasiparticles forms a Wigner crystal. As this crystal is topologically trivial, insulating, and compressible, it does not contribute to transport, so quantized Hall resistance is retained or restored at the value associated with the FHS (51).

This mechanism has been invoked to explain reentrant (fractional) quantum Hall in 2D electron gases (49, 50, 57), but for the present system we have two complicating factors. First, the presence of a moiré potential might be expected to favor forming a Wigner crystal specifically at rational fractional fillings of the moiré. We instead observe sustained quantization over a range of density that would encompass many simple fractions. Second, this plateau occurs in a regime of two valley-degenerate Hofstadter subbands, with bandwidth comparable to Zeeman splitting. There could thus be more than one way for the multiple flavors to combine into a fractional state, reminiscent of ν=2/3 FQH in a semiconductor double quantum well (58). If the electronic solid picture we suggest is correct, disentangling the precise nature of the solid, including whether it is partially or entirely composed of a second flavor of the composite fermions themselves, will be an important challenge.

Whether the uniaxial and biaxial heterostrain present in the sample we study enhances or suppresses FHS formation is an open question. Uniaxial strain strongly broadens the parent Chern subbands, blurring the intricate fractal nature of the Hofstadter spectrum (44, 47), as shown in *SI Appendix*, Fig. S25 where we compare the spectrum calculated within a strain-free model with versions incorporating strain. This enhanced dispersion of the parent Chern bands might have been expected to compete against electron interactions, suggesting strain could disfavor FHSs. On the other hand, FHSs have not been reported in TBG at similar twist angles, perhaps due to a sensitivity to strain in realizing FHSs or because high-field Landau fan measurements require access to national-scale facilities.

We return finally to the gapped states (B and C). Our model suggests that, even without electron–electron interactions, gapped ground states with t=0 may exist in which the net spin of all filled bands is nonzero. The configuration of both (B and C) is analogous to that of a zero-field quantum spin Hall (QSH) insulator, with not one but two pairs of counterpropagating edge modes. We might therefore expect a quantized longitudinal resistance $R_{\mathrm{xx}}=h/4e^{2}$ together with zero Hall resistance. Though the Hall resistance is indeed near zero at (B), the longitudinal resistance is strongly suppressed rather than quantized, so we cannot prove that some or all of these states in our experiment are QSH-like. If they indeed are QSH-like, our measurements suggest that the edge modes do not enter the ohmic contacts that serve as voltage probes, because of the narrowness of the arms connecting to the contacts and/or the multiple densities and thus fillings in series en route to the contacts. In this scenario, the voltage read out could be an average of the electrochemical potential of ballistically propagating left-going and right-going modes, and thus could be almost identical at successive contacts.

Our measurements reveal FHSs that persist over large regions of density and flux deep in the Hofstadter regime. Clarifying the true nature of these fractional states will require additional samples and measurements alongside further theory. We hope our observations motivate such efforts.

## Supplementary Material

Appendix 01 (PDF)

## Data Availability

All data and analysis code from this study are available at the Stanford Digital Repository (https://purl.stanford.edu/rs629jy3007) (59).

## Footnotes

^*^Here we reserve the term FCI for the context in which it was originally coined—fractionally quantized states in topological bands at zero magnetic field—which does not apply to the measurements discussed here. Instead, we use fractional Hofstadter state to denote an interaction-driven insulating state with fractionally quantized Hall resistance arising upon partial filling of a Hofstadter subband. Some recent literature refers to such states as FCIs.

^†^The standard approach to remove the effect of mixing is to antisymmetrize the longitudinal resistivity and symmetrize the Hall resistance as a function of magnetic field. Antisymmetrization could help reveal well-quantized plateaus in other Hall pairs. However, given the time constraints of our measurement run at the National High Magnetic Field Lab we were unable to acquire data for opposite field polarity.
