# Supplementary material for: Extended fractional Hofstadter states at high field in twisted bilayer graphene above the magic angle
Source: Proc Natl Acad Sci U S A. 2026 Oct 5;123(41):e2602080123. doi: 10.1073/pnas.2602080123 (PMC13663781; doi:10.1073/pnas.2602080123)
Supplement: Supplementary file 1 [file pnas.2602080123.sapp.pdf]

PNAS

Supporting Information for

Extended Fractional Hofstadter States at High Field in Twisted Bilayer Graphene Above the Magic Angle

Joe Finney, Aaron Sharpe, Linsey K. Rodenbach, Jian Kang, Xiaoyu Wang, Kenji Watanabe, Takashi Taniguchi, Marc A. Kastner, Oskar Vafek, and David Goldhaber-Gordon

Corresponding authors: Aaron Sharpe, Marc A. Kastner.
E-mail: aaron.sharpe@stanford.edu, mkastner@mit.edu

This PDF file includes:

Supporting text
Figs. S1 to S29
SI References

Supplemental material

S1. Transport measurements. We measured the sample in the SCM-32T system at the National High Magnetic Field Laboratory (MagLab). Although the hybrid magnet had a nominal maximum field of 32 T, as early users of the system we were restricted to a maximum field of 28 T. We used the top-loading dil fridge insert with a base temperature of 50 mK at the mixing chamber plate. The sample probe had 16 DC measurement wires, so we were not able to measure every contact pair in our device. The probe did not feature cold low-pass filters. We included low-pass filters (borrowed from another user and described in Ref. (1)) at the breakout box, however there was still RF pickup between the breakout box and the sample.

In addition to RF noise-induced blurring, our measurements also suffered from low-frequency noise within the measurement instruments themselves. This noise appeared in the current measurement for Fig. 2 (Fig. S7), but not in the associated voltage measurements for all densities/fields, meaning that the noise was not in the current itself. Thus, for Fig. 2, we do not use the current measurement, but instead divide by a constant 1 nA.

We sourced 1 nA of current at near 5 Hz using an SR860, sourcing one volt across a 1 GΩ resistor. We used SR550 and 560 voltage preamplifiers and SR830 and SR860 lock-in amplifiers to measure voltages. We used an Ithaco 1211 current preamplifier and an SR860 to measure current.

We took the first set of measurements on many contact pairs at once (Figs. S5 and S6). The Hall measurements in Fig. 2 were taken in this set. We then found that only measuring one contact pair at a time greatly reduced noise. We therefore took the measurement in Fig. 1, consisting only of a single longitudinal resistivity measurement, separately.

We show a comparison between the data taken at MagLab with data taken in our own system at Stanford in Fig. S3. The system at Stanford has a lower base temperature and high quality filters at the mixing chamber stage. There are two stages of filtering: the wires are first passed through a cured mixture of epoxy and bronze powder to filter GHz frequencies, then low-pass RC filters mounted on sapphire plates filter MHz frequencies. As a result, the Landau fan appears much sharper. The resistivity near charge neutrality is nearly an order of magnitude higher in our measurements at Stanford compared to those at MagLab.

There is one notable difference between how the measurements were performed at Stanford versus MagLab. At Stanford, our contacts became highly resistive when the graphite back gate was held at ground, so we fixed the graphite back gate to 1.5 V for the measurements. At MagLab, we found that the contacts were an order of magnitude lower resistance than what we saw at Stanford, so we left the graphite back gate at ground. The difference in contact resistances was likely a result of a difference in effective temperature between the two setups.

S2. Generalized Diophantine and Definitions. For concreteness, we explicitly follow the terminology used in (2), which we reproduce here. For a number of carriers per unit area $\bar{\rho}$, we write a generalized Diophantine equation as

$$\bar{\rho} = \nu_s \rho_\phi + \eta_s A_0^{-1}, \quad [1]$$

where ν_s and η_s are rational numbers, ρ_ϕ is the density of flux quanta per unit area, and A_0 is the unit cell area associated with the spatially modulated (crystalline) electron density. The Hall conductance is given by

$$\sigma_{xy} = -\nu_s e^2/h. \quad [2]$$

A Wigner crystal ($\eta_s \neq 0$, $\nu_s = 0$) will have a spatially modulated electron density but vanishing Hall conductance. An incompressible (fractional) quantum Hall liquid ($\eta_s = 0$, $\nu_s \neq 0$), and a Hall crystal ($\eta_s = 0$, $\nu_s \neq 0$) differ in that a quantum Hall liquid preserves translation symmetry whereas a Hall crystal spontaneously breaks translation symmetry with density fluctuations set by ρ_ϕ (an anomalous Hall crystal further spontaneously breaks time reversal symmetry). A Hall crystal differs from a Wigner crystal in having quantized Hall conductance: $\nu_s \neq 0$.

A partial Hall crystal ($\nu_s \neq 0$ and $\eta_s \neq 0$) is an "intermediate case" combining the properties of both a Wigner crystal and a Hall crystal (2). Therefore, it will have a non-zero Hall conductance and spatially modulated electron density. Given the Wigner crystal background, a partial Hall crystal will be compressible if the spatial modulation of the electron density is not strongly pinned to a disorder potential.

S3. Landau level reset at half flux. We observe a bending Landau minifan emitting from $n/n_s = 2$, $\Phi/\Phi_0 = 0.5$, where the $(0, 4)$ Středa line intersects half-flux (Fig. S1). In longitudinal transport (panel A), the minifan pointing toward higher density and higher flux bends non-monotonically back toward half flux. Such bending is also visible in the minifan pointing toward lower flux, but in the following discussion we focus on the more prominent upward-pointing minifan. There is a small kink where the bending Landau minifan would have intersected the $(1, 3)$ Středa line near $n/n_s = 2.5$, and a complete reset at $n/n_s = 3$. In Hall measurements (panel B), we observe $h/4e^2$ and $h/8e^2$ integer quantum Hall plateaus following the same bending behavior, including a third reset near $n/n_s = 3.5$ (see also Fig. S16).

This reset behavior is not the same bending behavior noted in Ref. (3) and cannot be understood in a non-interacting model of rigid bands. It is reminiscent of the Landau fan resets at integer filling in magic-angle twisted graphene structures (4–13). One possible explanation of this reset behavior in magic-angle samples is spontaneous isospin polarization. However, isospin polarization is inconsistent with our observation of a nonlinear trajectory in the space of filling and flux along which longitudinal resistivity dips and Hall resistance is quantized; an incompressible Chern gap must follow the appropriate Středa relation.

For each of the four combinations of spin and valley, the two zero-field moiré minibands split into four magnetosubbands at half flux, reflecting a unit cell doubling in the Hofstadter spectrum. Because of this unit cell doubling, integer filling of the highest-energy magnetosubband (two valley-degenerate subbands split by Zeeman) corresponds to fillings $n/n_s = 2.5$, 3, 3.5, and 4 referenced to the original unit cell. At $n/n_s = 3$, if the carriers within this band fully polarize into half of the available flavors, we expect a total Chern number of all

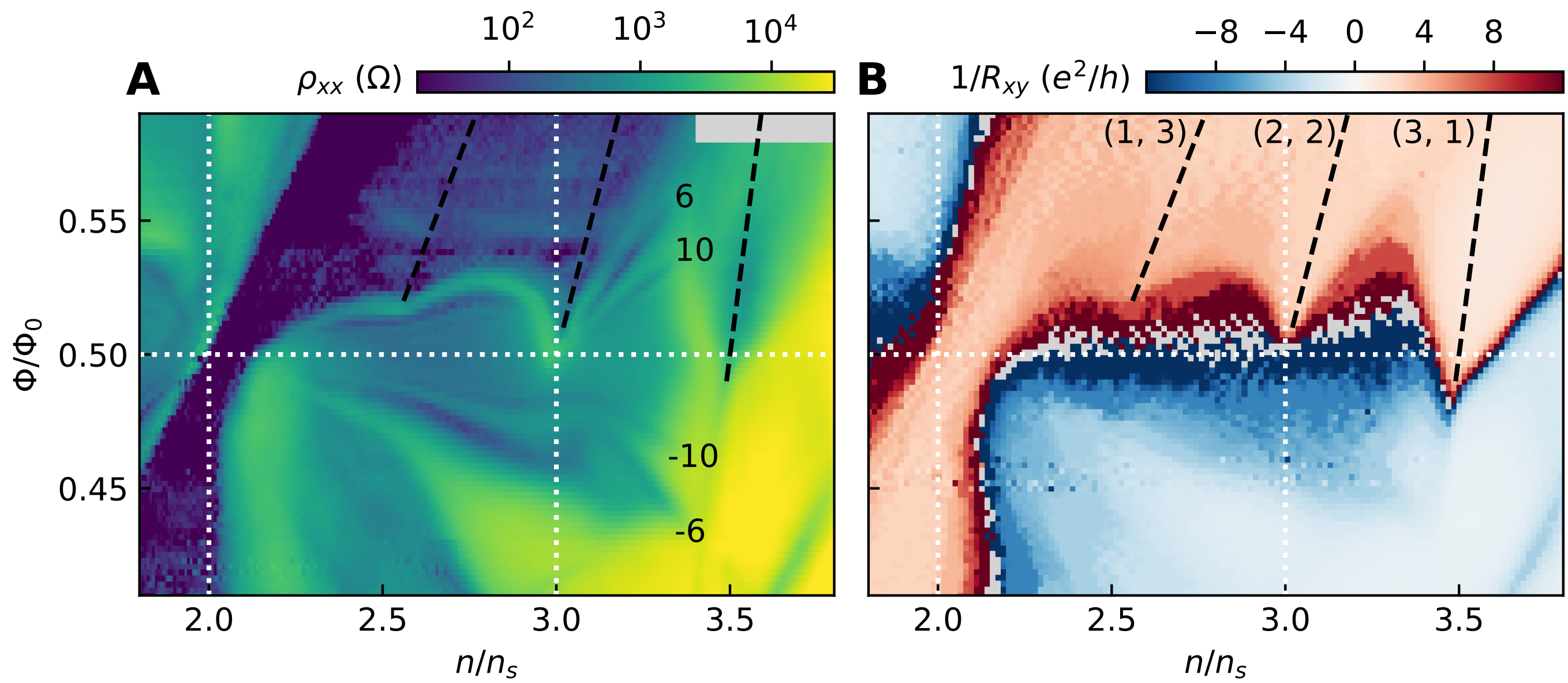

Fig. S1. Landau level reset at half flux. (**A**) Zoomed segment of Fig. 1 showing the Landau levels bending and resetting at half flux. The numbers ± 6 and ± 10 are the slopes (t values) of the faint lines $(3, \pm 6)_{1/2}$ and $(3, \pm 10)_{1/2}$ to their left. (**B**) Inverse Hall resistance showing that quantization of the Hall resistance tracks the bending dips in longitudinal resistivity. Detail of all contact pairs in Figs. S9, S10, and S11.

filled bands of 2. One can see this from the fact that this gap must be the same as the (2, 2) Hofstadter subband ferromagnet state emanating from zero field (13, 14). Landau levels emanating from half flux carry an extra two-fold degeneracy (see Sec. S8), therefore we would expect a four-fold degenerate fan with an offset of 2 ($t = \pm 2, 6, 10, \ldots$) emanating from $n/n_s = 3$. In our measurements, the Landau levels emanating from the reset point are indeed fourfold degenerate, with $(3, \pm 6)_{1/2}$ and $(3, \pm 10)_{1/2}$ being the only visible gaps. Here, we use the subscript 1/2 to indicate we are using s to reference a density offset at half of a flux quantum per moiré unit cell.

The resets do not all appear at exactly half flux. Rather, the reset seen in both longitudinal and Hall measurements near $n/n_s = 2.5$ is just above half flux, and the reset seen in Hall at $n/n_s = 3.5$ is just below half flux. Each reset aligns with an extrapolation of a Hofstadter ferromagnetic state—$(1, 3)$, $(2, 2)$, and $(3, 1)$, respectively—from zero field (dashed lines).

S4. Brown-Zak oscillations. At rational values of $\Phi/\Phi_0 = p/q$, the system can be described in terms of Bloch states in a q-times-enlarged magnetic Brillouin zone (15, 16). In transport, this manifests as oscillations in conductivity at simple field fractions and constant density. These "Brown-Zak" oscillations have been observed in graphene/hBN superlattices(17–19). The homogeneity and amount of disorder in the sample affects the maximum q for which oscillations will be visible, because as q increases, the size of the magnetic Brillouin zone increases relative to the carrier mean free path (18).

We observe Brown-Zak oscillations at $\Phi/\Phi_0 = 1/3$, 2/5, 1/2, and 3/5 (Fig. S4 panel B, horizontal dotted lines). Of note, the Brown-Zak oscillations at 1/2 flux appear only between $n/n_s = \pm 2$. The amplitude of such oscillations depends on the carrier group velocity and therefore the miniband width (18), so absence of Brown-Zak oscillations in certain bands may indicate that those bands are flatter, with correspondingly greater influence of electron interactions. In general, we observe more and stronger Brown-Zak oscillations on the electron side of charge neutrality compared to the hole side, consistent with the familiar observation that in TBG the hole bands are flatter than electron bands.

We observe a similar set of Brown-Zak oscillations in contact pairs 16-17, 7-8, and 17-18 (Fig. S5). Contact pair 6-7 has blurrier features overall, with Brown-Zak oscillations visible only at $\Phi/\Phi_0 = 1/2$ and 1/3. Contact pair 13-14 does not show clear oscillations, likely because of both spatial inhomogeneity and stronger interactions brought by closer proximity to the magic angle.

S5. Středa line fit procedure. The raw data for Figs. 1, S5, and S6 were collected as a function of gate voltage and magnetic field. We transform the data into the space of n/n_s vs Φ/Φ_0 by placing points along sharp lines with known (s, t) by-eye. Brown-Zak oscillations additionally constrain Φ/Φ_0. We then use least squares to fit parameters m_g, b_g, and B_0, where $n/n_s = m_g V_g + b_g$ and $\Phi/\Phi_0 = 1$ at $B = B_0$. The resulting fit parameters are shown in Table S1 and schematically with our device geometry in Fig. S2. In this process we neglect the role of quantum capacitance, which depends on density of states and thus in turn on carrier density, making any global linear conversion between gate voltage and density imperfect.

After fitting the global conversion parameters to lines with known (s, t), we were able to assign (s, t) to the remaining straight-line features. Most of the lines could be easily assigned by eye, and the results for all contact pairs are shown in Fig. S5. Not every relatively straight line in the space of density and flux along which ρ_{xx} has a local minimum corresponds to a quantum Hall Středa line, as already noted in Ref. (3). In Figs. S4 and S5 we attempted to label only Středa lines.

	m_g (V^{-1})	b_g	B_0 (T)	A (nm^2)	θ (°)
13 - 14	0.843 ± 0.005	1.171 ± 0.009	41.2 ± 0.4	100.4 ± 0.9	1.309 ± 0.006
6 - 16	0.723 ± 0.003	1.023 ± 0.006	48.0 ± 0.3	86.1 ± 0.5	1.414 ± 0.004
6 - 7	0.716 ± 0.002	0.990 ± 0.005	48.2 ± 0.2	85.8 ± 0.3	1.416 ± 0.002
16 - 17	0.774 ± 0.002	1.082 ± 0.004	45.1 ± 0.1	91.7 ± 0.2	1.369 ± 0.002
7 - 17	0.772 ± 0.002	1.081 ± 0.004	45.2 ± 0.2	91.5 ± 0.3	1.372 ± 0.003
7 - 8	0.771 ± 0.002	1.093 ± 0.003	45.1 ± 0.1	91.8 ± 0.1	1.369 ± 0.001
7 - 8*	0.770 ± 0.001	1.085 ± 0.002	45.2 ± 0.1	91.6 ± 0.1	1.371 ± 0.001
17 - 18	0.769 ± 0.002	1.096 ± 0.004	45.6 ± 0.1	90.8 ± 0.3	1.377 ± 0.002
8 - 18	0.734 ± 0.007	1.041 ± 0.010	47.6 ± 0.7	86.8 ± 1.3	1.408 ± 0.011

Table S1. Voltage to density conversion parameters. Fits to the data, primarily from Fig. S5. The * marks a second measurement of contact pair 7 - 8 only, from Fig. 1 – we were able to decrease measurement noise by measuring only this one contact pair. It is important that m_g and B_0 agree between the two measurements of contact pair 7 - 8, and they do. On the other hand, b_g may vary between cooldowns as charges in the dielectrics or at interfaces migrate. In this table we report twist angles calculated assuming zero strain, despite the known presence of ~ 0.2% uniaxial and biaxial heterostrain in the sample. At finite twist angle, moiré cell area depends on heterostrain only to second order in strain amount, but the influence of strain is magnified by the small twist angle (20). Neglecting uniaxial heterostrain biases our extracted twist angle by 0.001 degrees, comparable to our reported fit uncertainties. The effect of 0.2% biaxial heterostrain is substantially larger, biasing our extracted twist angle by 0.003 degrees, comparable and in some cases larger than our reported fit uncertainties. The unit cell area depends on biaxial homostrain to first order in strain amount, but without the small-angle magnification granted by heterostrain. Should biaxial homostrain be present in the sample at a level comparable to the heterostrain, neglecting it would bias our extracted twist angle by 0.001 degrees.

S6. Other contact pairs. Fig. S2 shows a schematic of the device with measured twist angles overlaid. We can roughly identify where in the device twist angle is inhomogeneous, based on the sharpness of transport features and the spatial variation of measured twist angle. For instance, contact pairs 6 - 16 and 6 - 7 have high measured twist angle together with very blurry features in transport.

Hence, we draw the blue region. Contact pairs 17 - 18 is slightly blurry, and has a slightly higher twist angle than its neighbors, but contact pair 8 - 18 is very blurry and has a significantly higher twist angle, hence the orange region. These regions are only our best estimates. We do not have any local probes to measure them directly.

Significant differences between neighboring contact pairs has the unfortunate consequence that analyses that rely on comparing two measurements, typically R_{xy} and ρ_{xx}, are not trustworthy.

S7. Single-particle bends, SBCI, Hofstadter ferrogmagnetism. As demonstrated in Ref. (3), not all relatively straight lines in field/density with low resistance are Středa gaps. Instead, relatively straight-line features of low resistivity can appear where one anisotropy-smeared butterfly spectrum is coincident with another. These false Středa lines have a characteristic behavior at intersections with lines from the other doubled butterfly. Many of the features within our measurements follow the same pattern, and we attempt to only place lines in Fig. S4B where we are confident that there is a gap.

We observe a few Středa lines with fractional $s = \pm 1/2$ and $t = \pm 3$ near half flux (Fig. S4 red dotted lines in panel B and right panels of Fig. S5). As they appear to emit from $n/n_s = \pm 2$ at half flux, we would describe them as $(\pm 2, \pm 3)_{1/2}$. They do not have a slope of 8/3 (Fig. S8). Such lines have been described as symmetry-broken Chern insulators in the literature (21), and we observe at least one of them in all of the contact pairs that we measured. Curiously, in contact pair 16 - 17, a symmetry-broken Středa line $(13/3, -4) = (3, -4)_{1/3} = (7/3, -4)_{1/2}$ runs between one third and one half flux.

We observe states with $s + t = \pm 4$ projecting from integer s (green dashed lines in Figs. S4 and S5). In all contact pairs, they tend to extend to lower field on the hole side compared to the electron side (Fig. S5). In samples from the literature near the magic angle, they extend to zero or near zero field (5, 6, 8–13). These lines are described theoretically as correlated Hofstadter ferromagnetic states in Ref. (22), meaning they have polarized spin and valley degrees of freedom of interaction-modified bands.

S8. Detail of Landau level reset. Figs. S9, S10, and S11 magnify the bending Landau minifan near $n/n_s = 2$, $\Phi/\Phi_0 = 1/2$ from Figs. 1, S5, and S6.

In Fig. S9, red dotted lines mark bending, low-resistance states. The green dashed $(1, 3)$ line is not visible as a minimum in ρ_{xx}. Instead, we include it to highlight that there is a kink in the red dotted lines where it would intersect them. This line corresponds to an integer filling of the band at half flux. After the reset at $n/n_s = 3$ (half filling of the band), we observe Středa lines $(3, \pm 6)_{1/2}$ and $(3, \pm 10)_{1/2}$.

The fourfold LL degeneracy is consistent with fully polarizing half of the bands at half flux. We note that above the topmost dotted red line, the transport behavior is the same as the spin-up regions described in the main text and Fig. S27. We suspect, therefore, that the band at half flux has fully spin-polarized at $n/n_s = 3$.

To show that the Landau levels emanating from half flux are two-fold degenerate (per spin per valley), the simplest way to see this is to look at the directly at the Hofstadter spectrum. For example, when crossing from the (4, -4) to the (0, 4) gap slightly above or below half flux, we cross a single Hofstadter subband (in the absence of Zeeman splitting). This corresponds to a total change in the Chern number of 8, so a single Landau level must carry an extra two-fold degeneracy.

To show this more concretely, we can start by considering a square lattice for simplicity. The effect of a magnetic field on the translation operators can be incorporated through a Peierls substitution

$$T_x = e^{i(p_x + eA_x)x/\hbar},\ T_y = e^{i(p_y + eA_y)y/\hbar} \quad [3]$$

where A is the vector potential for some choice of gauge. For a given flux through the unit cell Φ/Φ_0, these

operators will satisfy the commutation relation:

$$T_x T_y = T_y T_x e^{i2\pi\Phi/\Phi_0} \qquad [4]$$

In the case where the flux per unit cell is a rational fraction $\Phi/\Phi_0 = p/q$, for co-prime integers p and q, this commutation relation can be used to show

$$T_x^q T_y = T_y T_x^q; \qquad [5]$$

commutativity is restored over the enlarged magnetic unit cell.

If we specifically consider the Landau gauge and consider a specific wavefunction ψ_{n,p_y} that is an eigenstate of both the Hamiltonian and T_y. While this state is also an eigenstate of T_x^q, it is not an eigenstate of T_x. Therefore, there must exist some energetically degenerate state $T_x\psi_{n,p_y}$ with a different value of p_y. A state (or small cluster of states) that defines a low energy pocket of a band from which a sequence of Landau levels would form must be q-fold degenerate. In the case of half-flux, this means that a sequence of Landau levels has an extra two-fold degeneracy compared to a typical sequence emanating from zero field.

S9. Cluster filtering. The unfiltered data from Fig. 2 can be seen in Fig. S17. To filter the data, we begin by identifying points where the Hall resistance R_{xy} falls within some window of our desired value ($\pm1\%$ of $\pm3h/8e^2$ in this case). Binary dilation is applied to the windowed data such that nearest-neighbors within this masked array are now included. Each contiguous cluster of the dilated data set is then identified, and, if it does not contain above a threshold number of points, the original undilated points are omitted from the final filtered data set. Because of the dilation and generous threshold, this filtering primarily serves to remove isolated data points with no next-nearest-neighbor point within the window.

S10. How well quantized is the Hall resistance?. Fig. S15 shows histograms of $R_{xy} = V_{xy}/(1\ \text{nA})/g$ near the indicated ν values. Quantum Hall plateaus show up as sharp peaks in this histogram. The free parameter g represents inaccuracies in the gains of our amplifier and current source, which we did not measure carefully at MagLab. Then, we compute a metric of quantization

$$\delta = \frac{\text{argmax}_R - R^*}{R^*} \qquad [6]$$

for each ν value. This metric is simple and does not consider, for instance, shoulders in the histogram. We show values of δ computed for $g = 1.0785$. This number places $\nu = -8$, our most prominent quantum Hall plateau, directly on the expected value. It is instead possible to fine-tune g to 1.0824 so that all δ values are below 0.3%. Either way, we are confident in stating that our fractions are quantized to better than 1%. Note that 1.08 is a little high compared to our experience: typically the voltage pre-amps that we use at Stanford are only miscalibrated by a few percent. The 1.08 number also includes a factor from our current source, which we expect to be 1 to 2 percent based on the average of our noisy current (Fig. S7).

The simple δ metric does not consider the finite resolution of the voltage measurements: note that the histogram bins are typically spaced by a few tens of ohms. Assuming that it is σ_{xy} that is quantized with

$$\sigma_{xy} = \frac{\nu e^2}{h} = \frac{R_{xy}}{R_{xy}^2 + \rho_{xx}^2}, \qquad [7]$$

it is natural to ask how high can ρ_{xx} be for R_{xy} to still fall within the same histogram bin. For $\nu = \pm8/3$, the bins are roughly 20 Ω wide, yielding $|\rho_{xx}| \lesssim 300\ \Omega$. Indeed, in many of the places that R_{xy} is precisely quantized to $\pm8/3$, ρ_{xx} is of order a hundred ohms or less.

S11. Discussion of other fractions. In a similar manner to Fig. 2, in Fig. S19 we highlight where the Hall resistance R_{xy} falls within a 1% window about a specified fraction. We see that $\pm8/3$ is substantially more robust than the other displayed fractions. Most notably, $8/5$ and $-4/3$ exhibit the largest regions of quantization. Fractions not shown do not exhibit sizable regions that fall within a 1% window.

S12. Modeling a domain wall. To model the effects of a domain wall between two different Chern insulating regions with Chern numbers C_l and C_r, we can use the Landauer-Büttiker formalism,

$$I_i = \frac{e^2}{h}\sum_j (\bar{T}_{ji}V_j - \bar{T}_{ij}V_i), \qquad [8]$$

where the transmission coefficient $\bar{T}_{ij} = M_{ij}T_{ij}$ is the product of the number of modes M_{ij} between contact j to contact i, and the transmittance $0 \leq T_{ij} \leq 1$. We will consider the case of perfect edge modes with some probability t of a carrier initially starting in one edge mode ending in another. This will only have meaningful consequence for modes at the domain wall, where the carrier can end up in the adjacent domain. Let us first consider the case of a domain wall which passes in-between a Hall pair with $\text{sgn}(C_l) = \text{sgn}(C_r)$ (Fig. S21A). Assuming the source contact 4 is at voltage V and the drain contact 5 is at 0, for such a configuration, the full system of equations is

$$\begin{pmatrix} m_l & 0 & 0 & 0 & -m_l & 0 \\ -m_l & m_l & 0 & 0 & 0 & 0 \\ 0 & -\bar{T}_{12} & m_l & -\bar{T}_{32} & 0 & 0 \\ 0 & 0 & 0 & m_r & 0 & -m_r \\ 0 & 0 & -m_l & 0 & m_l & 0 \\ 0 & -\bar{T}_{15} & 0 & -\bar{T}_{35} & 0 & m_r \end{pmatrix} \times \begin{pmatrix} V \\ V \\ V_2 \\ 0 \\ V \\ 0 \end{pmatrix} = \begin{pmatrix} 0 \\ 0 \\ 0 \\ 0 \\ I \\ -I \end{pmatrix},$$

where $m_i = |C_i|$. Then

$$V_2 = T_{12}V,$$
$$I = m_l V(1 - T_{12}) = \bar{T}_{15}V.$$

We can then solve for the expected resistance values:

$$R_{xx}^{\text{bot}} = \frac{V_0 - V_1}{I} = 0$$
$$R_{xx}^{\text{top}} = \frac{V_2 - V_3}{I} = \frac{T_{12}}{m_l}\frac{1}{1 - T_{12}}$$
$$R_{yx}^{\text{left}} = \frac{V_0 - V_2}{I} = \frac{1}{m_l}$$
$$R_{yx}^{\text{right}} = \frac{V_1 - V_3}{I} = \frac{1}{m_l}\frac{1}{1 - T_{12}}.$$

We can similarly consider the case of a domain wall which passes in-between a Hall pair with $\mathrm{sgn}(C_l) \neq \mathrm{sgn}(C_r)$ (Fig. S21B). Again, assuming the source contact 4 is at voltage V and the drain contact 5 is at 0, for such a configuration, the full system of equations is

$$\begin{pmatrix} m_l & 0 & 0 & 0 & -m_l & 0 \\ -m_l & m_l & 0 & 0 & 0 & 0 \\ 0 & -\bar{T}_{12} & m_l & 0 & 0 & -\bar{T}_{52} \\ 0 & -\bar{T}_{13} & 0 & m_r & 0 & -\bar{T}_{53} \\ 0 & 0 & -m_l & 0 & m_l & 0 \\ 0 & 0 & 0 & -m_r & 0 & m_r \end{pmatrix} \times \begin{pmatrix} V \\ V \\ V_2 \\ V_3 \\ V \\ 0 \end{pmatrix} = \begin{pmatrix} 0 \\ 0 \\ 0 \\ 0 \\ I \\ -I \end{pmatrix}.$$

For this case,

$$\begin{aligned} V_2 &= T_{12}V, \\ V_3 &= T_{13}V, \\ I &= m_l V(1 - T_{12}). \end{aligned}$$

Solving for the expected resistance values:

$$\begin{aligned} R_{xx}^{\mathrm{bot}} &= \frac{V_0 - V_1}{I} = 0 \\ R_{xx}^{\mathrm{top}} &= \frac{V_2 - V_3}{I} = \frac{1}{m_l}\frac{T_{12} - T_{13}}{1 - T_{12}} \\ R_{yx}^{\mathrm{left}} &= \frac{V_0 - V_2}{I} = \frac{1}{m_l} \\ R_{yx}^{\mathrm{right}} &= \frac{V_1 - V_3}{I} = \frac{1}{m_l}\frac{1 - T_{13}}{1 - T_{12}} = \frac{1}{m_l}\frac{T_{12}}{1 - T_{12}}. \end{aligned}$$

For either case, it would be possible to achieve a measured Hall resistance of 3/8 with the appropriate values of m_l and T_{12}. However, such a scenario would yield markedly different values for R_{xx}^{bot} and R_{xx}^{top}, which is inconsistent with our data. Therefore, we think such a domain wall in our sample is not the case.

Following this same procedure, one can see that a domain wall that does not pass between a Hall pair cannot yield a fractionally quantized Hall conductance.

S13. Continuum Model and Quantum metric. In this subsection, we discuss the Hamiltonian of the continuum model in the presence of both heterostrain and out-of-plane magnetic field. Heterostrain is described by a 2×2 symmetric matrix S_ϵ which can always be diagonalized by an orthogonal matrix $R(\phi)$,

$$S_\epsilon = R_\varphi^T \begin{pmatrix} -\epsilon_{\mathrm{uni}} + \epsilon_{\mathrm{bi}} & 0 \\ 0 & \nu\epsilon_{\mathrm{uni}} + \epsilon_{\mathrm{bi}} \end{pmatrix} R_\varphi \,. \qquad [9]$$

where $R_\varphi = \begin{pmatrix} \cos\varphi & -\sin\varphi \\ \sin\varphi & \cos\varphi \end{pmatrix}$ is the two-dimensional rotational matrix with the angle of φ that determines the two principal axes of the strain matrix S_ϵ, and R_φ^T is the transpose of the matrix R_φ, $\nu = 0.16$ is the Poisson ratio as used in Ref. (20), and ϵ_{uni} and ϵ_{bi} are the uniaxial strain and biaxial strain respectively. In principle, the two layers may have different strain matrices $S_\epsilon^{(t)}$ and $S_\epsilon^{(b)}$, where the superscript t and b refers to top and bottom layers respectively. However, in this work, we consider only heterostrain with $S_\epsilon = S_\epsilon^{(t)} - S_\epsilon^{(b)}$ and neglect the homostrain $\frac{1}{2}(S_\epsilon^{(t)} + S_\epsilon^{(b)})$, since the shape of moire unit cell, the narrow band structure, the associated band properties, etc. are more sensitive to heterostrain whose impact is estimated as the maximal value of ϵ/θ with $\epsilon = \max(|\epsilon_{uni}|, |\epsilon_{bi}|)$ is the larger one between the uniaxial strain and the biaxial strain. On the other hand, the impact of homostrain is estimated as $\sim \epsilon$, much smaller than the heterostrain, and therefore can be safely neglected for small twist angles.

The impact of heterostrain on the non-interacting Hamiltonian have been discussed in Ref. (20, 23, 24), so we only briefly summarize the results in this manuscript. Following the model introduced in Ref. (23, 24), we recalculated the lattice relaxation in the presence of the heterostrain by iteration method. We found that the change of the relaxation by the applied heterostrain is the order of ϵ/θ. To achieve the precision of 1 meV in the dispersion of the narrow bands and include the particle-hole breaking terms, our continuum model contains all the terms up to the second order in the gradient expansion, with all the parameters numerically calculated from the microscopic tight binding model proposed in Ref. (23, 24). The Hamiltonian can be divided into two parts, the intralayer Hamiltonian H_{intra} and the interlayer tunneling H_{inter}. The presence of heterostrain introduced several additional terms in H_{intra}. In addition to the coupling between the pseudo-scalar field induced by lattice distortion and the fermion density, H_{intra} also includes the couplings between various strain-induced fields and the stress-energy of the Dirac fermion. H_{inter} includes both the contact and gradient couplings. Since the tunneling constants, such as w_0, w_1, and w_2 in BM model, depends on both the microscopic interlayer hopping and the in-plane lattice distortion, and thus also depends on the applied strain. We have numerically calculated these coefficients with their values presented in Ref. (24, 25).

As illustrated in Ref. (20), the presence of heterostrain, in general, breaks the three-fold rotation C_3 and C_{2x} symmetry, but still conserves $C_2\mathcal{T}$. As a consequence, the two Dirac cones around the CNP are not gapped, but the Dirac points are shifted to other momenta in the moiré Brillouin zone, and the two Dirac points are not degenerate anymore, with the energy difference manipulated by heterostrain and observable by quantum oscillation experiments.

Crucially, the three degenerate van Hove points become non-degenerate as uniaxial heterostrain is introduced. In Ref. (20), we measured the density of these non-degenerate van Hove points for both electrons and holes. Our model was electron/hole symmetric, so we could not fit to all six van Hove points simultaneously. Here, given we have now incorporated e-hole asymmetry, we can.

Fig. S26 shows the χ^2 statistic for a wide variety of moire parameters ϵ_{uni}, ϵ_{bi}, and ϕ. Given these three free parameters, we choose θ such that the moire unit cell area is fixed. In practice, fixing θ instead to 1.37° does not make a significant change to these results.

There are two minima in ϵ_{bi}, at -0.5% and 0.3%. These two minima correspond to the same Hamiltonian after accounting for symmetries, with their asymmetry about zero ϵ_{bi} coming from the Poisson ratio (which converts uniaxial heterostrain into both uniaxial heterostrain and a small amount of biaxial heterostrain). Thus, there is a unique set of parameters yielding a best fit.

In Ref. (20), we estimated an uncertainty of roughly $\delta\nu = \pm 0.05$ on each van Hove point. Here, our best-fit result does satisfy this bound on each van Hove

point. We hesitate to place uncertainties on our best-fit parameters, because we believe that they will be dominated by systematics. The model is not perfect, and it is not straightforward to account for model uncertainty. The uncertainties that we do report are based on the width of the minima in Fig. S26, and are underestimates.

The fits are only performed using low-field data. We find that at high field, the computed results look qualitatively similar for the amount of strain estimated in Ref. (20) and the amount fit here. See Fig. S25.

The Hamiltonian in the presence of a magnetic field can be obtained by substituting the operator $\hat{\boldsymbol{p}} \longrightarrow \hat{\boldsymbol{p}} + e\boldsymbol{A}(\boldsymbol{x})$, with the Landau gauge $\boldsymbol{A} = B(0, y)$.

For each field p/q, the computation produces a list of energies, eigenvalues of some large Hamiltonian. The number of energies produced is not the same from one field to another, growing with q. We then add a Zeeman term $E = \pm g\mu_B B/2$ for $g = 2$ and μ_B is the Bohr magneton. Fig. S22A shows the resulting spectrum.

To convert the list of energies at a given field E_i into something that we can plot versus density, we follow Ref. (26) and write

$$\rho(E) = \sum_i \frac{1}{\pi} \frac{\gamma}{(E - E_i)^2 + \gamma^2},$$

$$n(E) = \sum_i \frac{1}{\pi} \arctan\left(\frac{E - E_i}{\gamma}\right).$$

To normalize, divide each equation by the number of energies. The smoothing parameter γ is not based on any temperature or disorder model. Fig. S22B is then $1/\rho(E)$ plotted against $n(E)$ and field. It is refreshing to see that even with such a simple model, much of the behavior from both Ref. (3) and the present manuscript are captured.

At the end of this subsection, we briefly review the definition of Berry connection and quantum metric for the Bloch states. As usual, the overlap of two Bloch states are

$$\begin{aligned}\langle u(\boldsymbol{k})|u(\boldsymbol{k}+\delta\boldsymbol{k})\rangle &= 1 + \delta k_\mu \langle u(\boldsymbol{k})|\partial_\mu u(\boldsymbol{k})\rangle \\ &+ \frac{1}{2}\delta k_\mu \delta k_\nu \langle u(\boldsymbol{k})|\partial_\mu \partial_\nu u(\boldsymbol{k})\rangle + O(\delta k^3)\end{aligned} \qquad [10]$$

where $u(\boldsymbol{k})$ is the perodic part of the corresponding Bloch state, i.e. $u(\boldsymbol{k}) = e^{-i\boldsymbol{k}\cdot\boldsymbol{r}}\phi(\boldsymbol{k})$, where $\phi(\boldsymbol{k})$ is the Bloch state with the crystal momentum of $\boldsymbol{k}$. Here, the overlap $\langle u(\boldsymbol{k}+\delta\boldsymbol{k})|u(\boldsymbol{k})\rangle$ is expanded to the quadratic order of $\delta\boldsymbol{k}$. It is obvious that the linear part of δk_μ is proportional to the Berry connection, defined as

$$\mathcal{A}_\mu = i\langle u(\boldsymbol{k})|\partial_\mu u(\boldsymbol{k})\rangle$$

and can be shown to be real. Additionally,

$$\langle u(\boldsymbol{k})|\partial_\mu \partial_\nu u(\boldsymbol{k})\rangle = -i\big(\partial_\mu \mathcal{A}_\nu + \partial_\nu \mathcal{A}_\mu\big) + \langle \partial_\mu \partial_\nu u(\boldsymbol{k})|u(\boldsymbol{k})\rangle \, . \qquad [11]$$

Therefore, the imaginary part of $\langle u(\boldsymbol{k})|\partial_\mu \partial_\nu u(\boldsymbol{k})\rangle$ is

$$-\frac{1}{2}\big(\partial_\mu \mathcal{A}_\nu + \partial_\nu \mathcal{A}_\mu\big) \qquad [12]$$

However, it does not appear in the quadratic terms of the expansion since it is an anti-symmetric tensor. We introduce the symmetric tensor $\gamma_{\mu\nu}$ for the real part

$$\gamma_{\mu\nu} = -\mathrm{Re}(\langle u(\boldsymbol{k})|\partial_\mu \partial_\nu u(\boldsymbol{k})\rangle) \qquad [13]$$

Thus, Eq. 10 can be rewritten as

$$\begin{aligned}&\langle u(\boldsymbol{k})|u(\boldsymbol{k}+\delta\boldsymbol{k})\rangle = 1 - i\mathcal{A}_\mu \delta k_\mu \\ &- \frac{1}{2}\delta k_\mu \delta k_\nu \left(\gamma_{\mu\nu} + \frac{i}{2}\big(\partial_\mu \mathcal{A}_\nu + \partial_\nu \mathcal{A}_\mu\big)\right) + O(\delta k^3)\end{aligned} \qquad [14]$$

However, the γ tensor is $U(1)$ gauge dependent. To obtain a gauge-independent tensor, consider

$$\begin{aligned}&|\langle u(\boldsymbol{k})|u(\boldsymbol{k}+\delta\boldsymbol{k})\rangle|^2 \\ &= 1 - \delta k_\mu \delta k_\nu \left(\gamma_{\mu\nu} - \mathcal{A}_\mu \mathcal{A}_\nu\right) + O(\delta k^3)\end{aligned} \qquad [15]$$

and thus, the quantum metric tensor is defined as

$$g_{\mu\nu}(\boldsymbol{k}) = \gamma_{\mu\nu}(\boldsymbol{k}) - \mathcal{A}_\mu \mathcal{A}_\nu \qquad [16]$$

$$\Longrightarrow 1 - |\langle u(\boldsymbol{k})|u(\boldsymbol{k}+\delta\boldsymbol{k})\rangle|^2 = \delta k_\mu \delta k_\nu g_{\mu\nu}(\boldsymbol{k}) + O(\delta k^3) \qquad [17]$$

In the presence of magnetic fields, the magnetic Bloch states might be written as the linear combination of Landau wave function instead of plane waves. In this case, the overlap between $u(\boldsymbol{k})$ and $u(\boldsymbol{k}+\delta\boldsymbol{k})$ is calculated as

$$\begin{aligned}&\langle u(\boldsymbol{k})|u(\boldsymbol{k}+\delta\boldsymbol{k})\rangle \\ &= \frac{1}{S_{\mathrm{uc}}} \int_{\mathrm{muc}} \mathrm{d}^2\boldsymbol{r}\ \phi^*_{\boldsymbol{k}}(\boldsymbol{r}) e^{-i\delta\boldsymbol{k}\cdot\boldsymbol{r}} \phi_{\boldsymbol{k}+\delta\boldsymbol{k}}(\boldsymbol{r})\end{aligned} \qquad [18]$$

where $\int_{\mathrm{muc}} \mathrm{d}^2\boldsymbol{r}$ integrates only over one magnetic unit cell, and $\phi_{\boldsymbol{k}}$ is the magnetic Bloch state with the magnetic momentum of $\boldsymbol{k}$.

We show the Berry curvature and the trace of the quantum metric tensor for the bands featuring $\pm 8/3$ FHSs at $2/5$ flux in Fig. S23. We note that the "trace condition" (27) is violated strongly in the case of one of these bands (panel B).

S14. Spin dependent transport detail. There is a curious asymmetry in the longitudinal transport measurements. Observe the value of the resistivity on either side of $(0, \pm 4)$ in Fig. 1. On the left side (lower density), the resistivity tends to be in the kilohms, and there are numerous Středa lines immediately next to the $(0, \pm 4)$ line (See Sec. S14 for more detail). On the right side (higher density), the resistivity tends to be lower than a kiloohm, and in some cases it is below one Ohm. In these regions, there are no Středa lines, and the resistivity is flat and featureless.

As an example, a line cut at constant field is shown in Fig. S27 panel A. To the left of $(0, 4)$, the resistivity is peaks around 4 kilohms. To the right, between $(0, 4)$ and $(2, 0)$, it is only a hundred Ohms or lower. In panel B we show that these two types of transport correspond to states in the model that are entirely spin-polarized. Where the states are spin-up, we see low resistivity, and where they are spin-down we see high resistivity. Where we see a mixture of both, the resistivity is even higher. This behavior is followed in our measurements not just

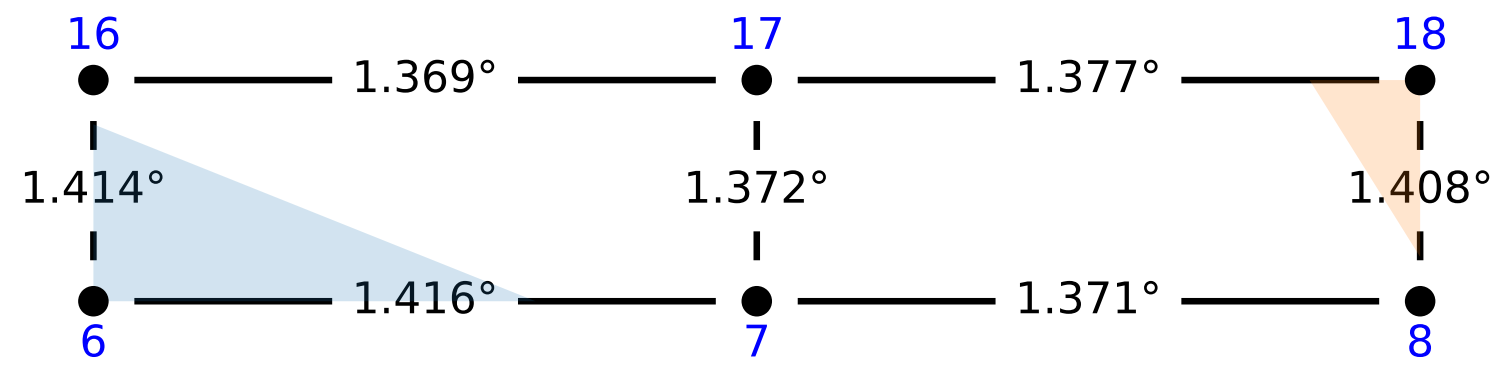

Fig. S2. Twist angle variation. Blue-labelled points are our contacts. The pitch between contacts is 3 μm. The channel is 1 μm wide. Vertical segments between them are Hall pairs, and horizontal segments are longitudinal pairs. The estimated θ values for each contact pair (Table S1) are laid over the lines, with uncertainties elided for brevity. Shaded regions indicate our guess of regions with particularly inhomogeneous twist angles, based on nearby twist angles and the sharpness of features in the transport measurements.

near $(0, \pm 4)$, but also in other regimes where the model reasonably matches the experiment. The major exception to this rule is near charge neutrality, where no substantial asymmetry is apparent.

Curiously, we only see FHSs in these low-resistivity (spin-up) regions. Circled α, β, and γ in Fig. 3 panels A and B indicate regions where we observe $\pm 8/3$ FHSs. All of these regions are within blue Chern bands.

Spin-dependent resistivity has been observed in quantum Hall systems previously (28–30). With Zeeman splitting, the lower-energy, spin-up states are filled first. Spin-down electrons then see a different magnetic environment: there is a substantial background of spin-up electrons already present. In high-quality WSe_2, this leads to the minority-spin resistance being dramatically increased at low temperatures (30). We observe the same behavior here, however we are not confident that the underlying explanation is the same. We may be able to reverse the direction of resistivity with back gate voltage. See Fig. S3 panels C and D from Ref. (3), where oscillations appear as a function of displacement field (y-axis). We wish to emphasize that we do not have positive evidence for the hypothesis of spin-dependent transport.

1. P Wang, et al., Piezo-driven sample rotation system with ultra-low electron temperature. Rev. Sci. Instruments **90** (2019).
2. Z Tešanović, F Axel, BI Halperin, "Hall crystal" versus Wigner crystal. Phys. Rev. B **39**, 8525–8551 (1989).
3. J Finney, et al., Unusual magnetotransport in twisted bilayer graphene. Proc. Natl. Acad. Sci. **119** (2022).
4. Y Cao, et al., Unconventional superconductivity in magic-angle graphene superlattices. Nature **556**, 43–50 (2018).
5. M Yankowitz, et al., Tuning superconductivity in twisted bilayer graphene. Science **363**, 1059–1064 (2019).
6. JM Park, Y Cao, K Watanabe, T Taniguchi, P Jarillo-Herrero, Tunable strongly coupled superconductivity in magic-angle twisted trilayer graphene. Nature **590**, 249–255 (2021).
7. D Wong, et al., Cascade of electronic transitions in magic-angle twisted bilayer graphene. Nature **582**, 198–202 (2020).
8. SL Tomarken, et al., Electronic compressibility of magic-angle graphene superlattices. Phys. Rev. Lett. **123** (2019).
9. J Yu, et al., Correlated hofstadter spectrum and flavour phase diagram in magic-angle twisted bilayer graphene. Nat. Phys. **18**, 825–831 (2022).
10. KP Nuckolls, et al., Strongly correlated chern insulators in magic-angle twisted bilayer graphene. Nature **588**, 610–615 (2020).
11. AT Pierce, et al., Unconventional sequence of correlated chern insulators in magic-angle twisted bilayer graphene. Nat. Phys. **17**, 1210–1215 (2021).
12. Y Xie, et al., Fractional chern insulators in magic-angle twisted bilayer graphene. Nature **600**, 439–443 (2021).
13. Y Saito, et al., Hofstadter subband ferromagnetism and symmetry-broken chern insulators in twisted bilayer graphene. Nat. Phys. **17**, 478–481 (2021).
14. X Wang, O Vafek, Theory of correlated chern insulators in twisted bilayer graphene. Phys. Rev. X **14** (2024).
15. E Brown, Bloch electrons in a uniform magnetic field. Phys. Rev. **133**, A1038–A1044 (1964).
16. J Zak, Magnetic translation group. Phys. Rev. **134**, A1602–A1606 (1964).
17. RK Kumar, et al., High-temperature quantum oscillations caused by recurring bloch states in graphene superlattices. Science **357**, 181–184 (2017).
18. RK Kumar, et al., High-order fractal states in graphene superlattices. Proc. Natl. Acad. Sci. **115**, 5135–5139 (2018).
19. J Barrier, et al., Long-range ballistic transport of brown-zak fermions in graphene superlattices. Nat. Commun. **11** (2020).
20. X Wang, et al., Unusual magnetotransport in twisted bilayer graphene from strain-induced open fermi surfaces. Proc. Natl. Acad. Sci. **120** (2023).
21. EM Spanton, et al., Observation of fractional chern insulators in a van der waals heterostructure. Science **360**, 62–66 (2018).
22. X Wang, O Vafek, Theory of correlated chern insulators in twisted bilayer graphene. Phys. Rev. X **14**, 021042 (2024).
23. O Vafek, J Kang, Continuum effective hamiltonian for graphene bilayers for an arbitrary smooth lattice deformation from microscopic theories. Phys. Rev. B **107** (2023).
24. J Kang, O Vafek, Pseudomagnetic fields, particle-hole asymmetry, and microscopic effective continuum hamiltonians of twisted bilayer graphene. Phys. Rev. B **107** (2023).
25. J Kang, O Vafek, Analytical solution for the relaxed atomic configuration of twisted bilayer graphene including heterostrain. Phys. Rev. B **112**, 125138 (2025).
26. K Hejazi, C Liu, L Balents, Landau levels in twisted bilayer graphene and semiclassical orbits. Phys. Rev. B **100** (2019).
27. PJ Ledwith, G Tarnopolsky, E Khalaf, A Vishwanath, Fractional chern insulator states in twisted bilayer graphene: An analytical approach. Phys. Rev. Res. **2** (2020).
28. K Vakili, et al., Spin-dependent resistivity at transitions between integer quantum hall states. Phys. Rev. Lett. **94** (2005).
29. D Maryenko, et al., Spin-selective electron quantum transport in nonmagnetic mgzno heterostructures. Phys. Rev. Lett. **115** (2015).
30. EM Shih, et al., Spin-selective magneto-conductivity in WSe2. Nat. Phys. **21**, 1231–1236 (2025).

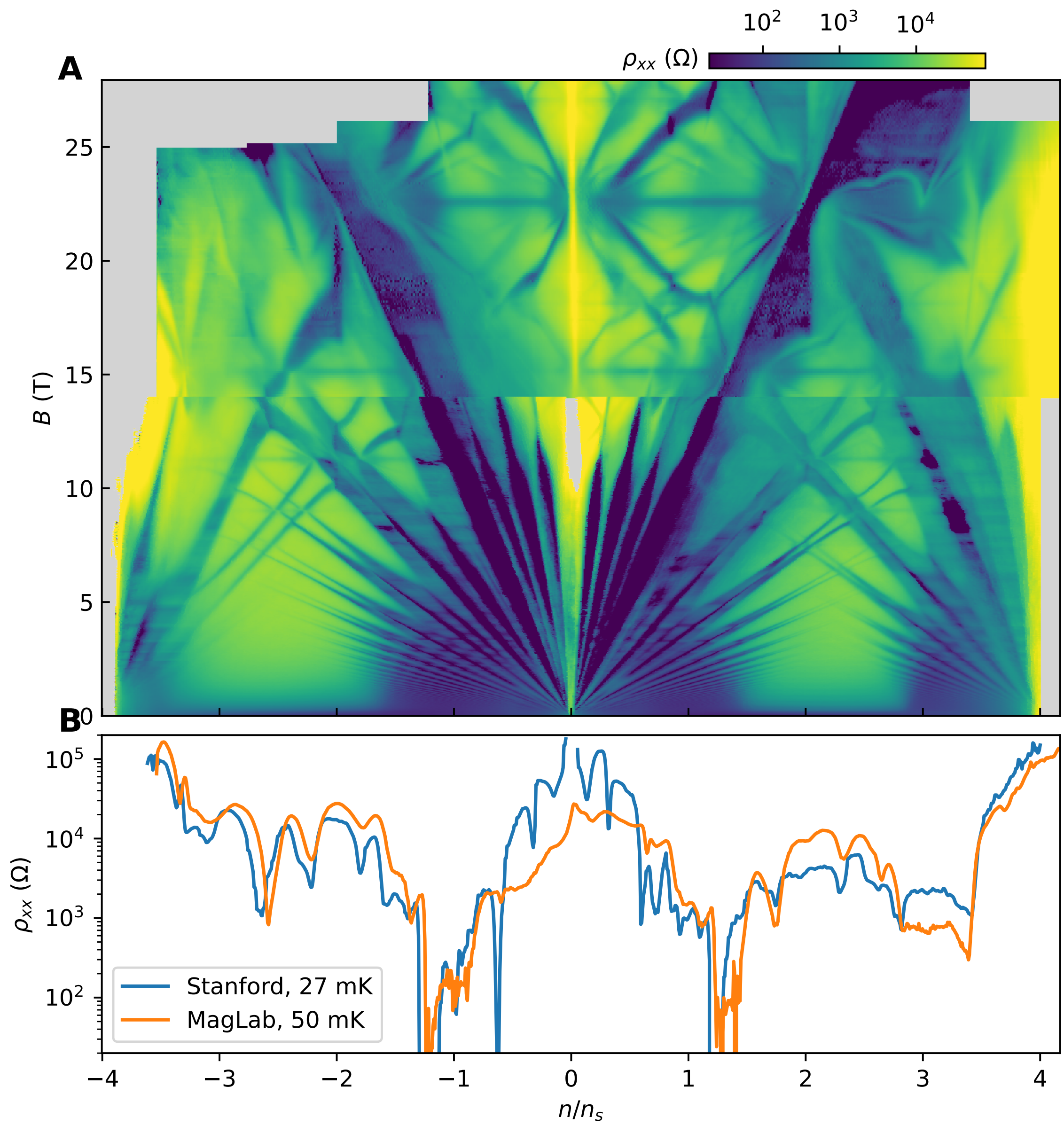

Fig. S3. Full Landau fan including data taken at lower field. Contact pair 7 - 8. (**A**) Data from 0 to 14 T are taken in our system at Stanford, previously published in Ref. (3). Data above 14 T are the same as in Fig. 1, taken at MagLab. (**B**) Line cuts from both systems at 14 T.

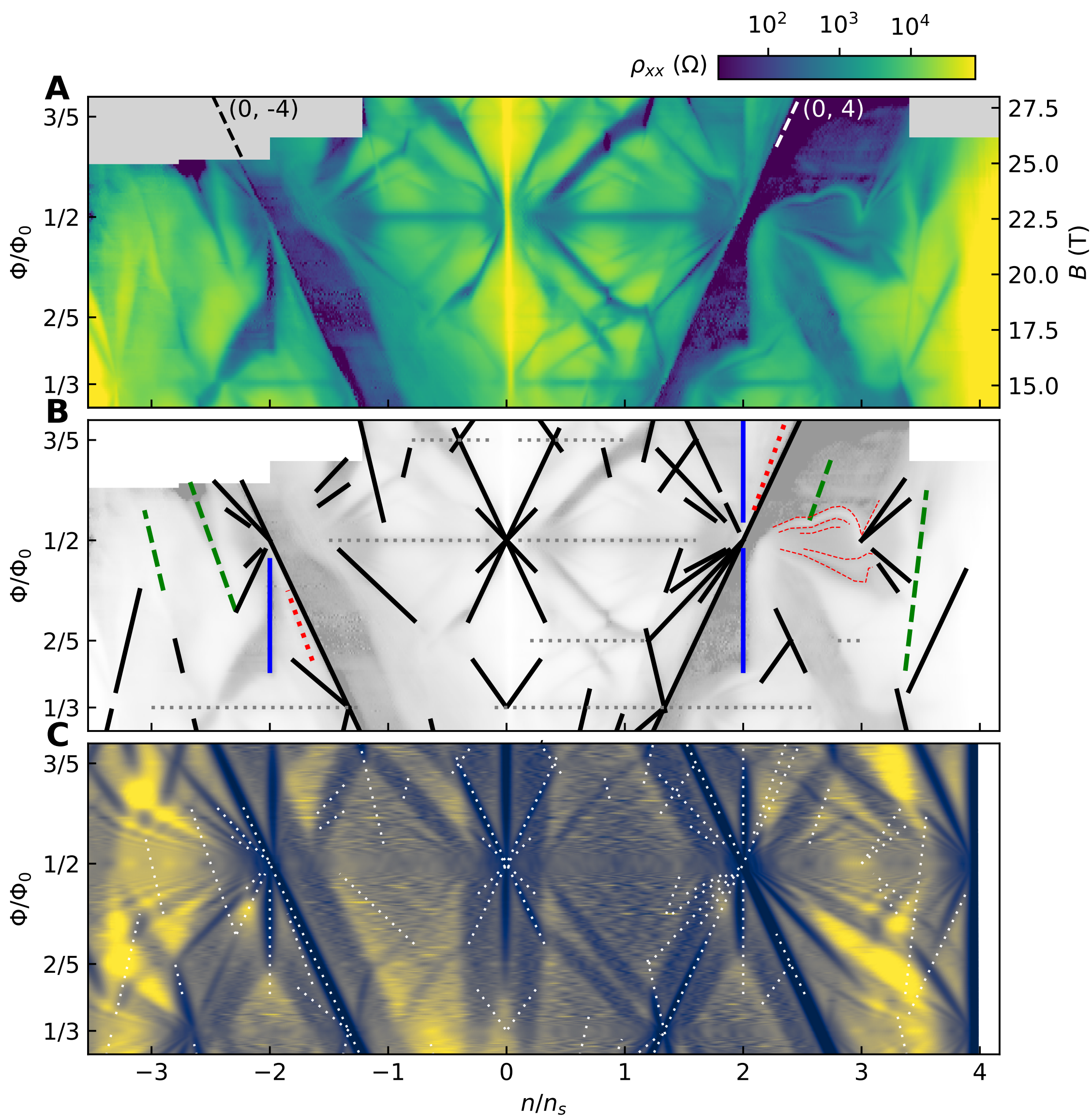

Fig. S4. Longitudinal fan with schematic. (**A**) Same data as in Fig. 1. (**B**) Black lines are ordinary integer quantum Hall. Blue vertical lines are potential QSH sites discussed in the main text. Green dashed lines are correlated Hofstadter ferromagnetic states (Sec. S7). Red dotted lines are symmetry-broken Chern insulating states (Sec. S7). Red dashed lines are the bending Landau levels discussed in Sec. S3. Gray dashed lines are Brown-Zak oscillations. (**C**) The solid black lines from B overlaid on the computation results as white dotted lines.

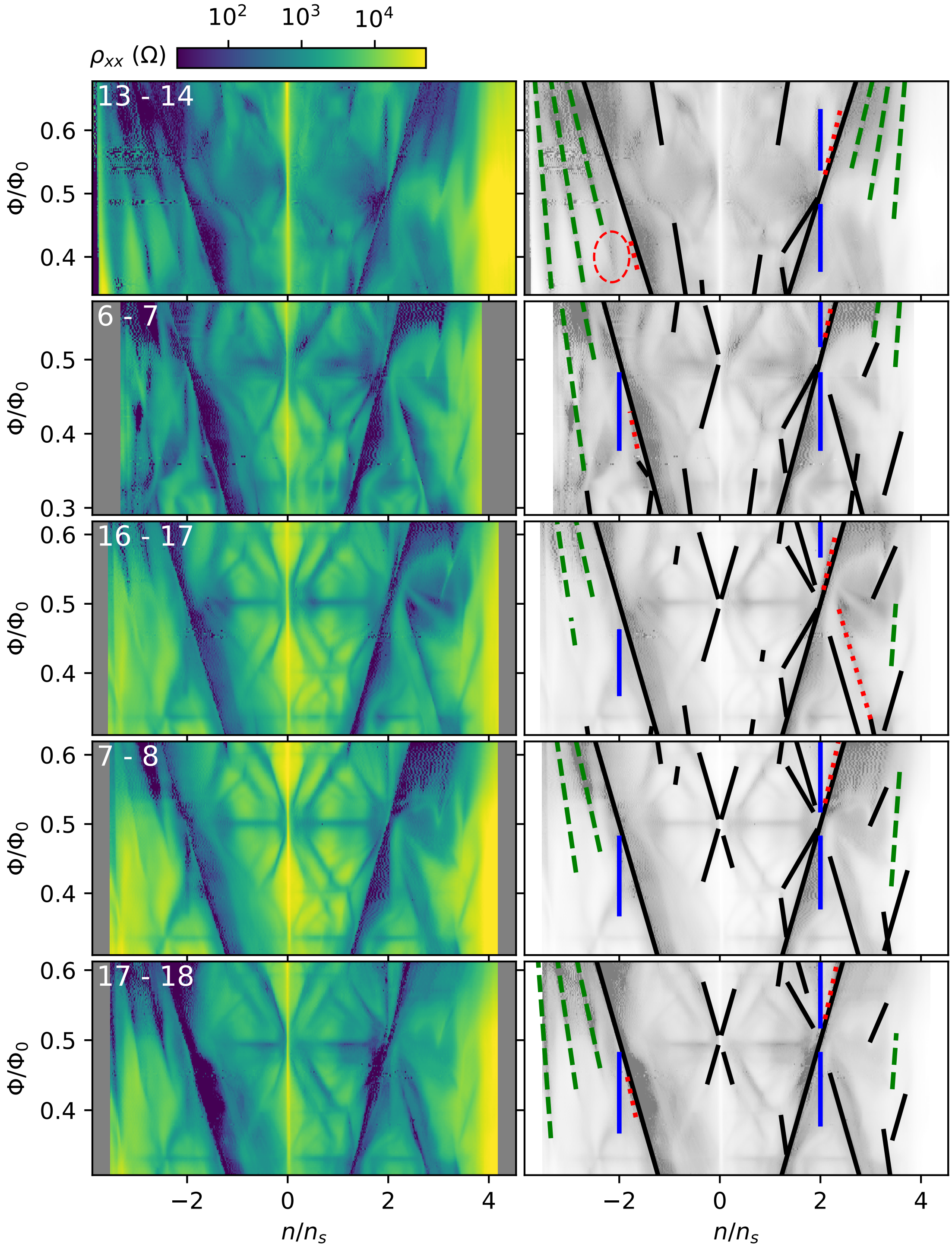

Fig. S5. All longitudinal contact pairs. Measured separately from Fig. 1, which was a remeasurement of contact pair 7-8. (**Left panels**) The longitudinal resistivity of the indicated contact pairs. (**Right panels**) Corresponding schematic with the same interpretation as in Fig. S4B. The red circle for contact pair 13 - 14 indicates a feature that we could not unambiguously identify. It does not appear to be (-1, -3), which can be seen above it.

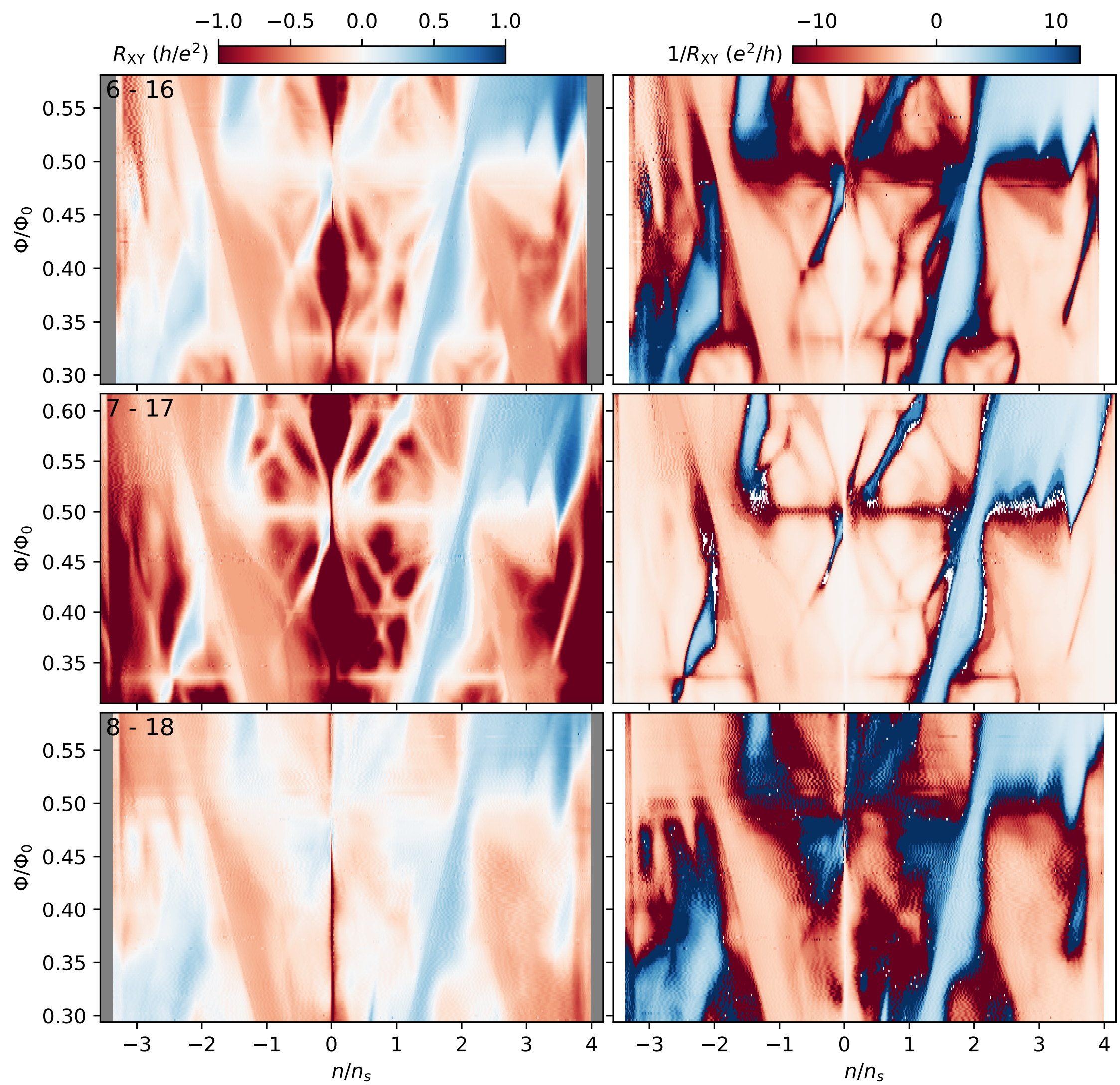

Fig. S6. All Hall contact pairs. (**Left panels**) R_{xy} for the indicated contact pairs. (**Right panels**) Corresponding $1/R_{\mathrm{xy}}$. Line cuts for each are shown in Fig. S14. Note that contact pairs 6 - 16 and 8 - 18 show significantly blurrier features compared to 7 - 17.

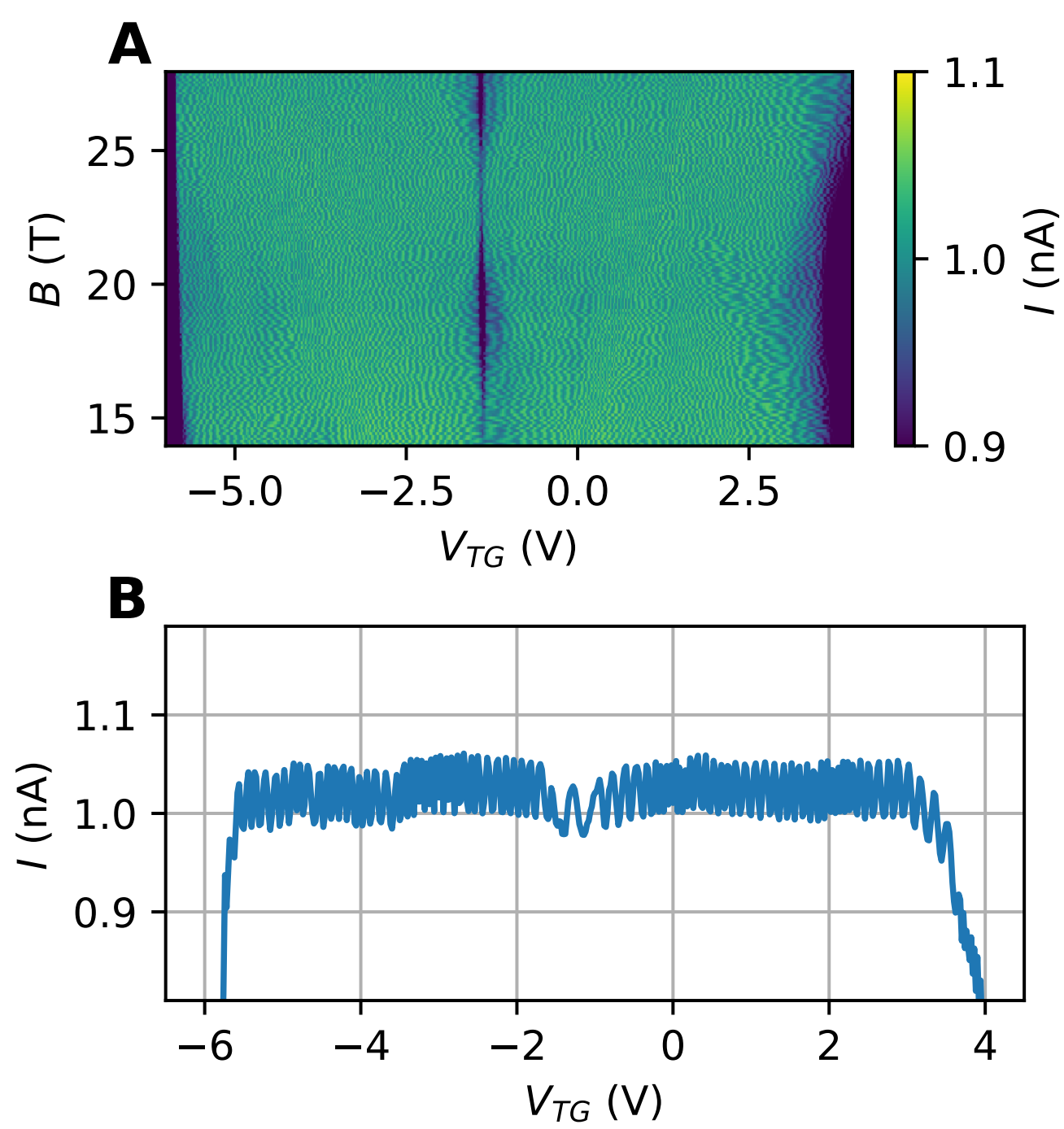

Fig. S7. Current noise. (**A**) Current vs gate and field. (**B**) Line cut at $B = 14$ T. Note that the average current is a few percent above 1 nA.

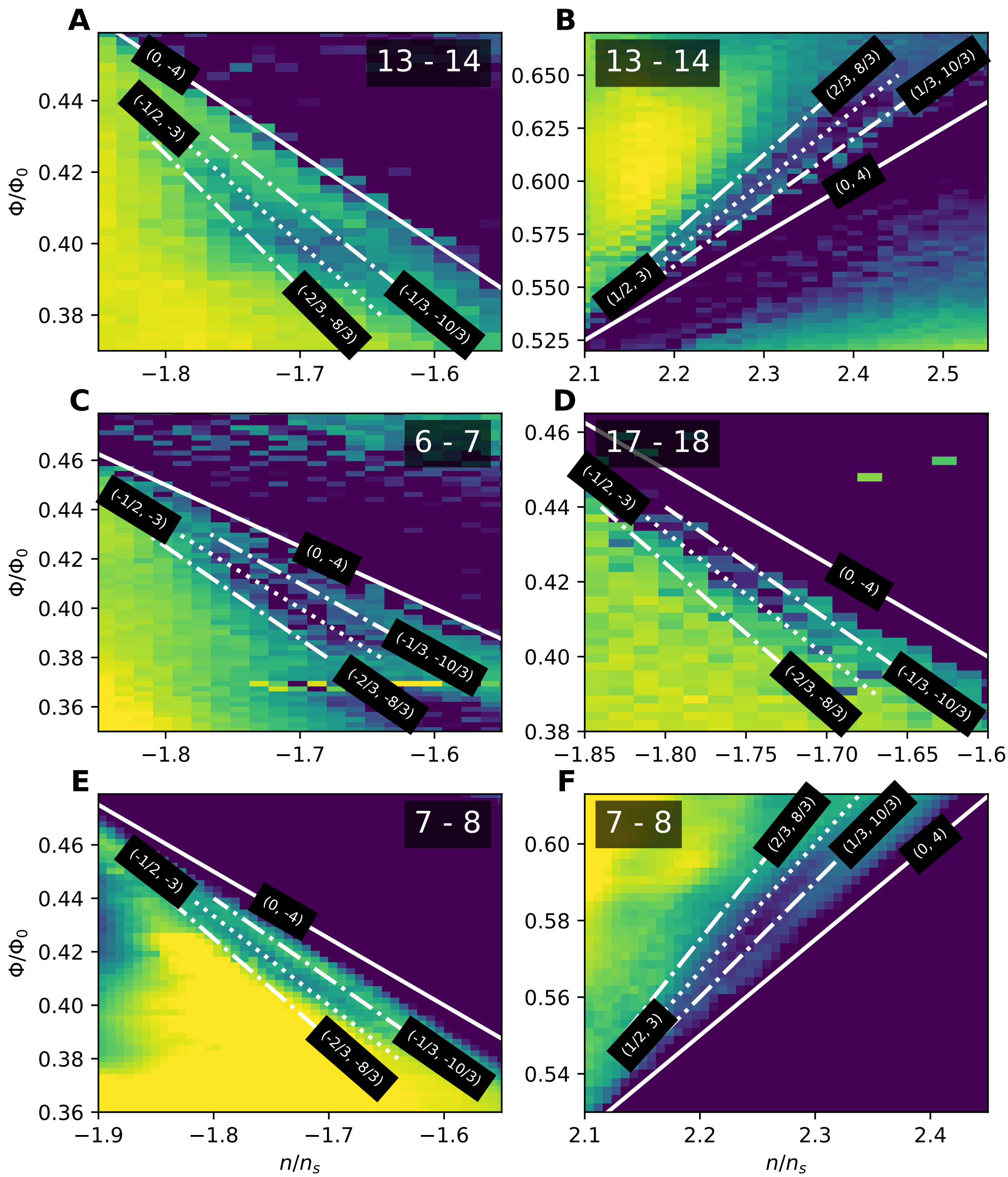

Fig. S8. Fractional or symmetry-broken Středa lines near half flux? Panels **A**-**D** are the same data as in Fig. S5. Panels **E**-**F** are the same data as in Fig. 1. Colormaps are adjusted for each panel to highlight the relevant line. Fits to $(\pm 1/2, \pm 3)$ (dotted lines) are better than fits to third fractions (dash dotted lines) in all cases except for panel **F**.

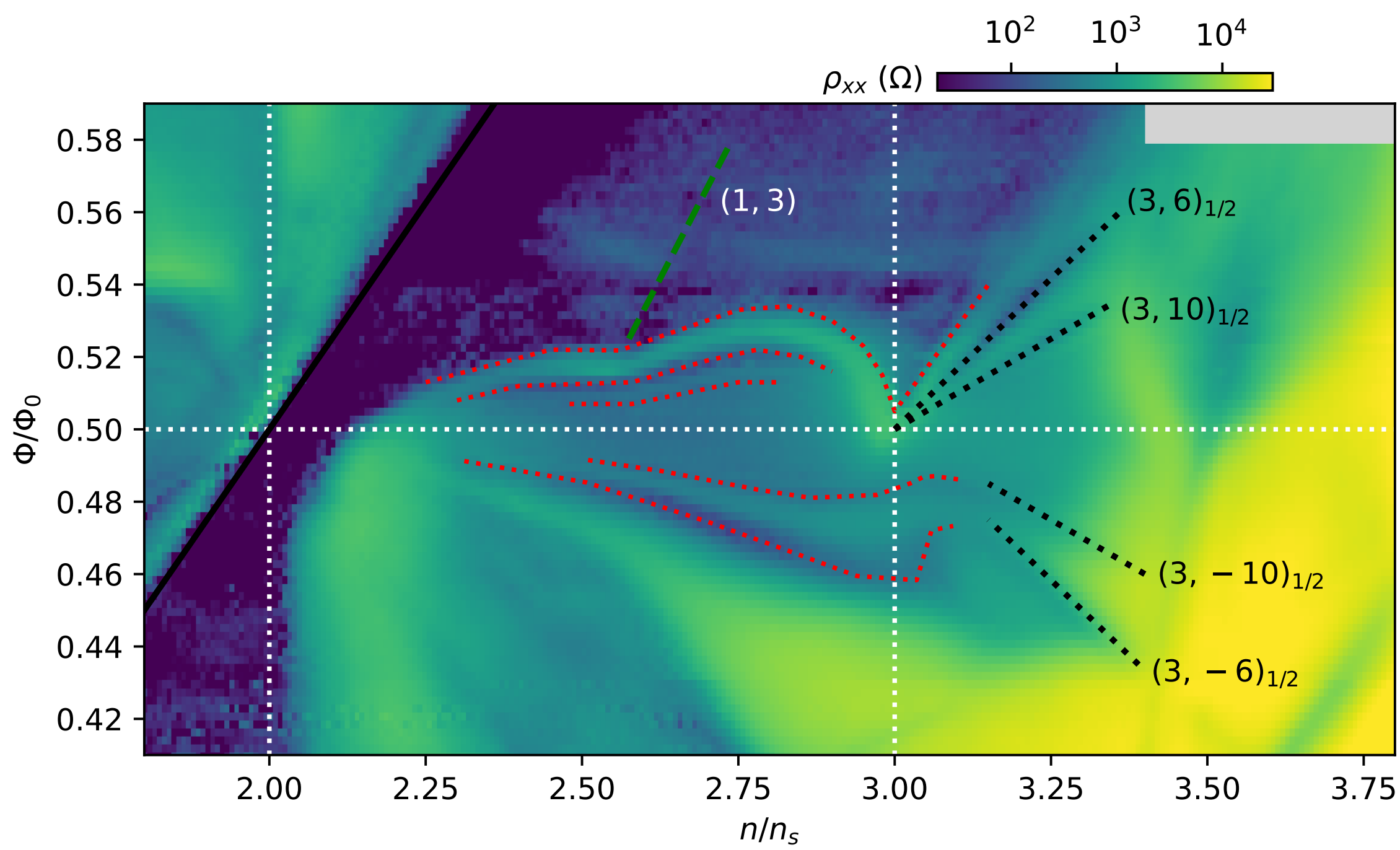

Fig. S9. Detail of LL reset. Data from Fig. 1. Other contact pairs shown in Figs. S10 and S11. The green dashed line $(1, 3)$ is not prominently visible in the resistivity, but lines up with the kink in the red dotted line below it. The red dotted lines indicate minima in ρ_{XX} and likely correspond to Landau level gaps. The black dotted lines are minima that follow the indicated paths

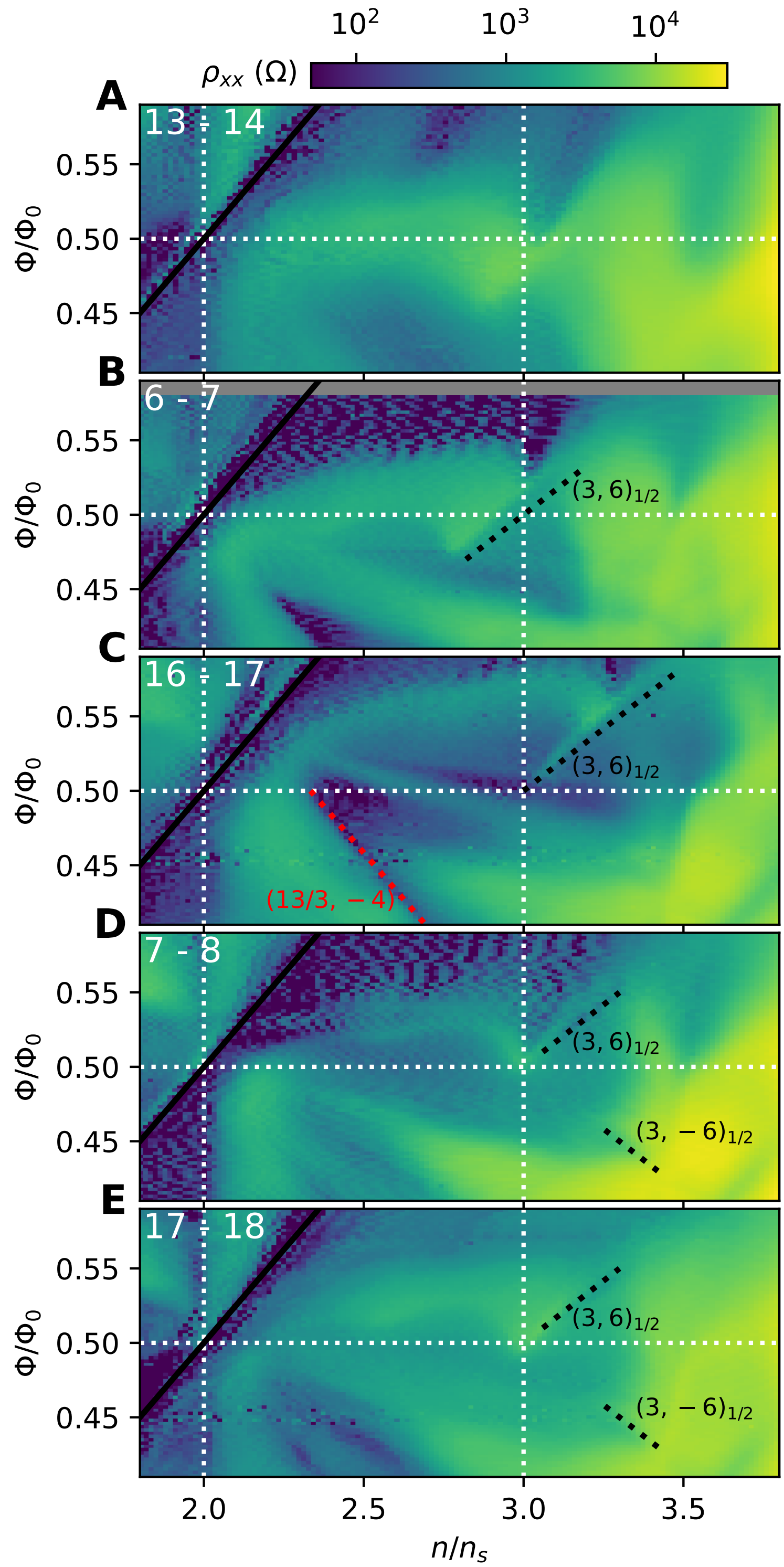

Fig. S10. Detail of LL reset, continued. All longitudinal contact pairs, labelled as in Fig. S9.

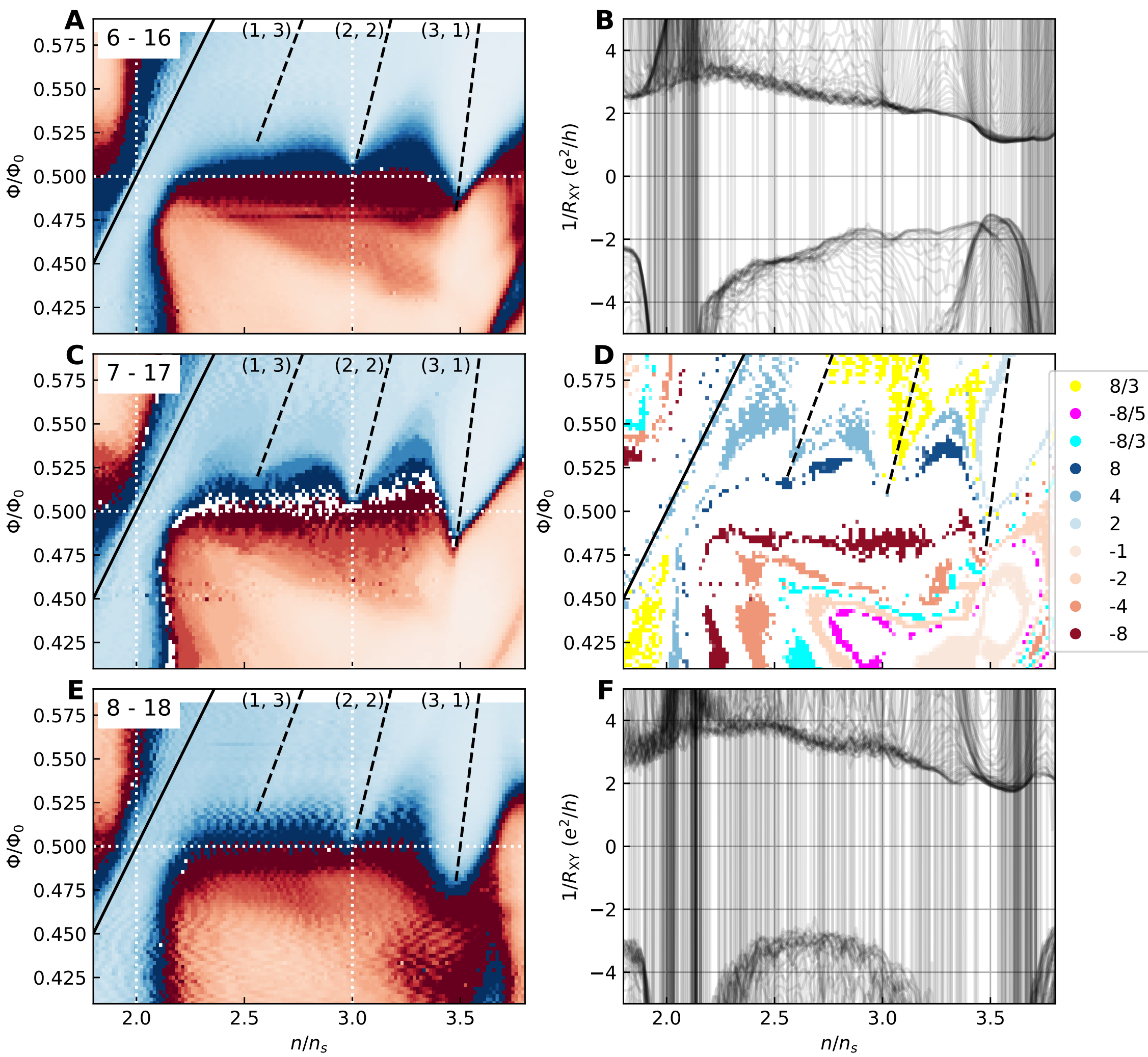

Fig. S11. Detail of LL reset, continued. All three Hall contact pairs show a change of sign near half flux (**ACE**). There appear to be features associated with the Středa lines $(1, 3)$, $(2, 2)$, and $(3, 1)$, however the Hall conductivity never has the expected value for them (**BDF**). The sign change along the $(2, 2)$ line happens very close to half flux and $n/n_s = 3$, however the sign change along $(3, 1)$ happens slightly below half flux in all cases.

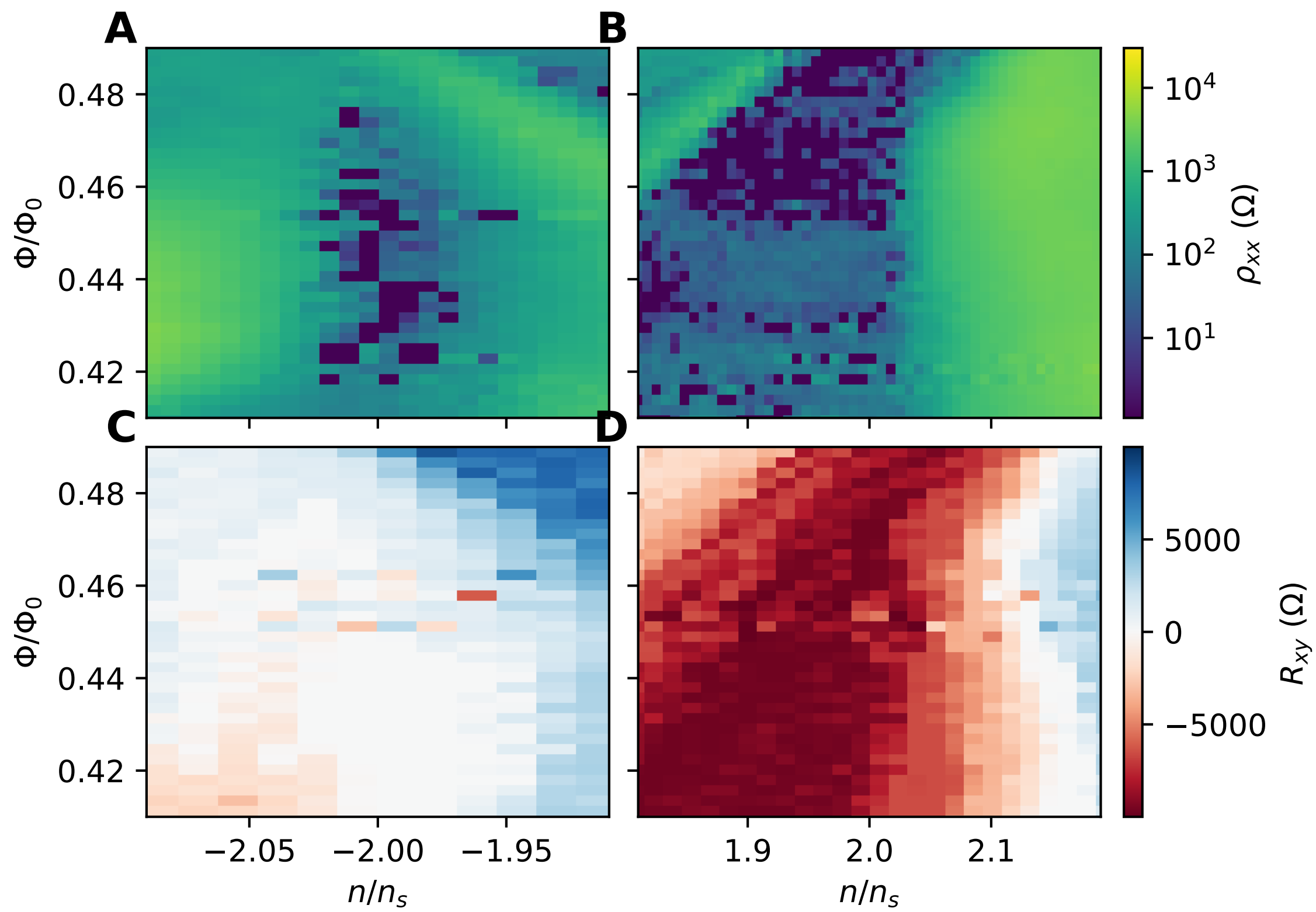

Fig. S12. Fine detail of potential QSH. (**A**-**B**) Zoom in on Fig. 1 near the clear vertical-line features. (**C**-**D**) Same, for contact pair 7 - 17.

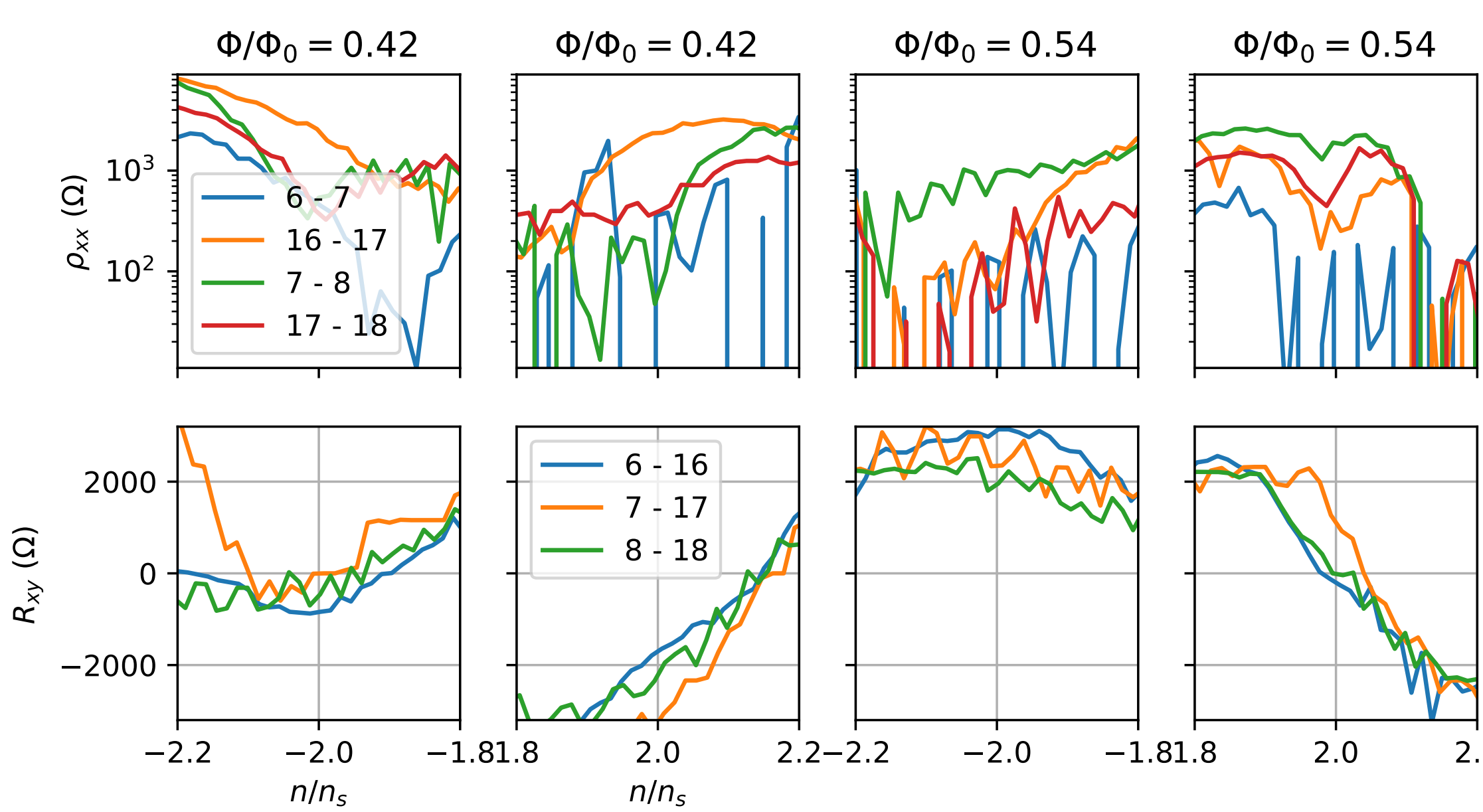

Fig. S13. Line cuts near potential QSH. Top row: line cuts of longitudinal measurements (Fig. S5) at the indicated fields. Although there are some minima at $n/n_s = \pm 2$, there is too much noise in most measurements to clearly claim a certain behavior. Bottom row: ditto, but for Hall measurements (Fig. S6). Only contact pair 7 - 17 shows a clear plateau at zero resistance at $n/n_s = -2$ and $\Phi/\Phi_0 = 0.42$.

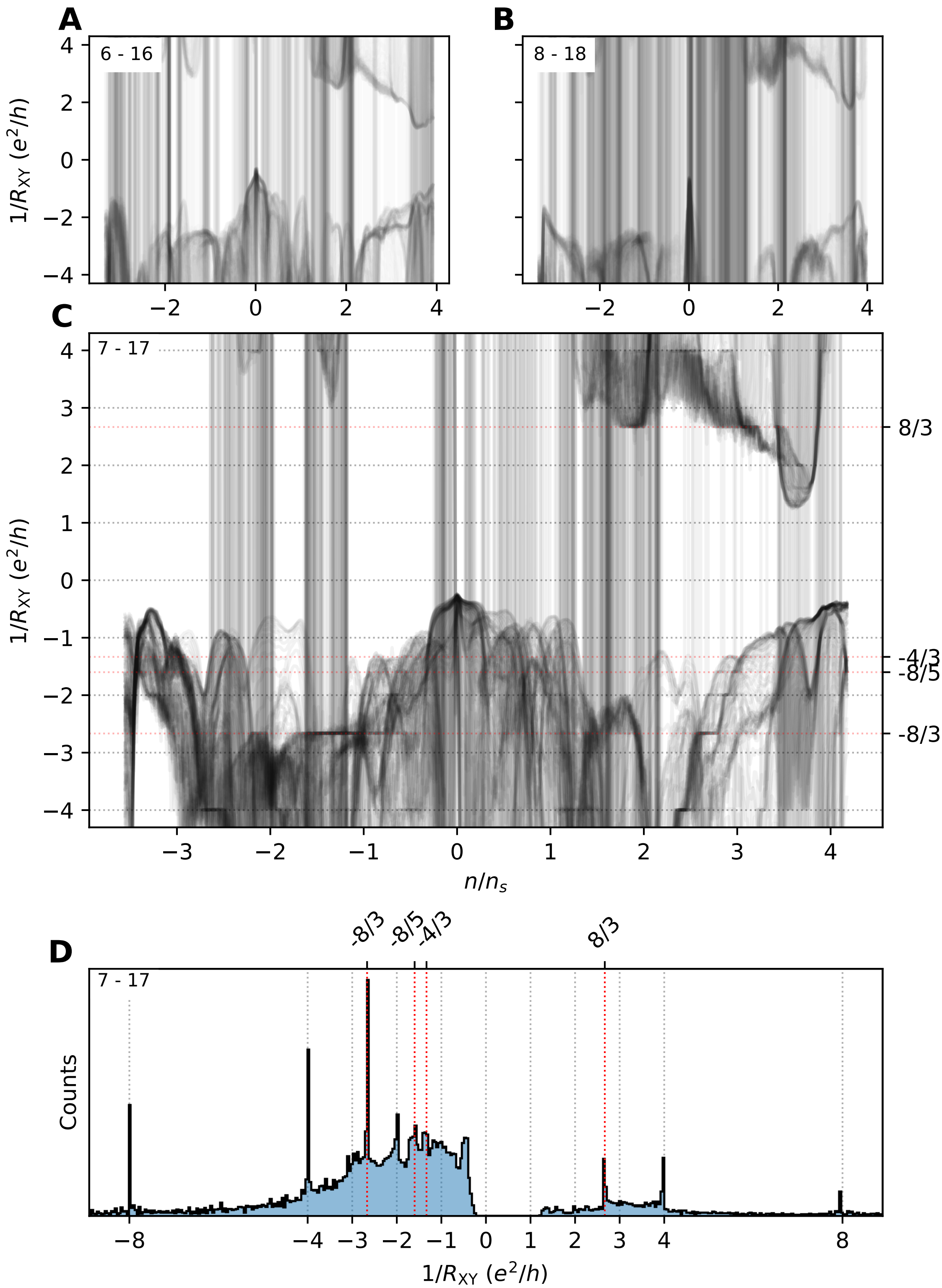

Fig. S14. Hall plateaus. All line cuts at constant field of a Landau fan for a given contact pair plotted over each other with transparency. Quantized Hall plateaus will appear as solid horizontal lines. Contact pairs indicated in the top left corner. (**A**, **B**) There are no quantized Hall plateaus for these contact pairs, not even integers. (**C**) Contact pair 7 - 17 shows quantized plateaus at integers ± 8 (not shown), ± 4, -2, -8/3, -8/5, and -4/3, the latter two only near $n/n_s = 3$. (**D**) Histogram of all Hall conductances from panel B. Interestingly, there are more -8/3 measurements than any integer.

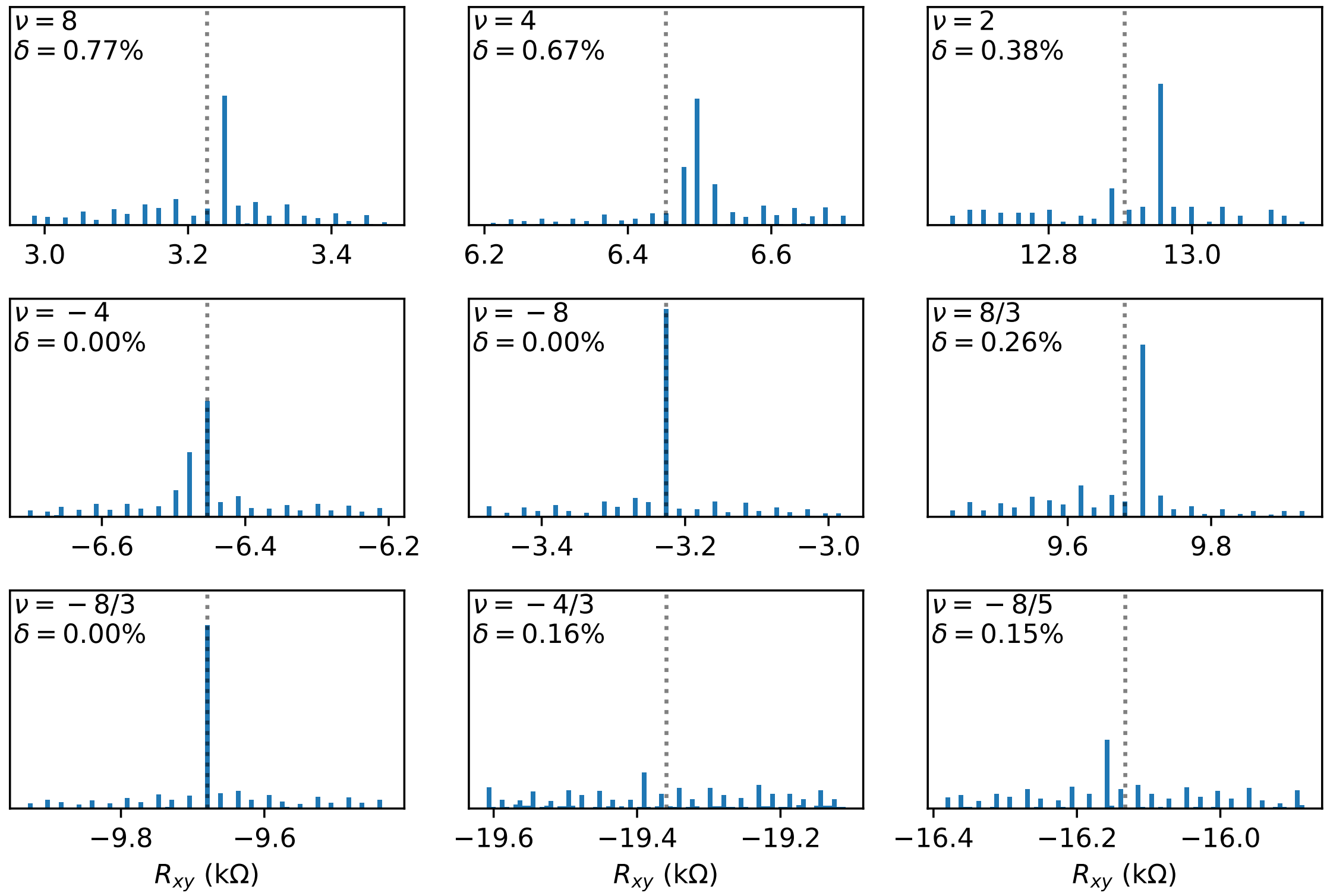

Fig. S15. How well quantized is the Hall resistance? Each panel shows a histogram (over the entire gate and field ranges) of R_{xy} centered around the expected value of $h/\nu e^2$ (vertical dotted line) for $g = 1.0785$ along with the computed δ value.

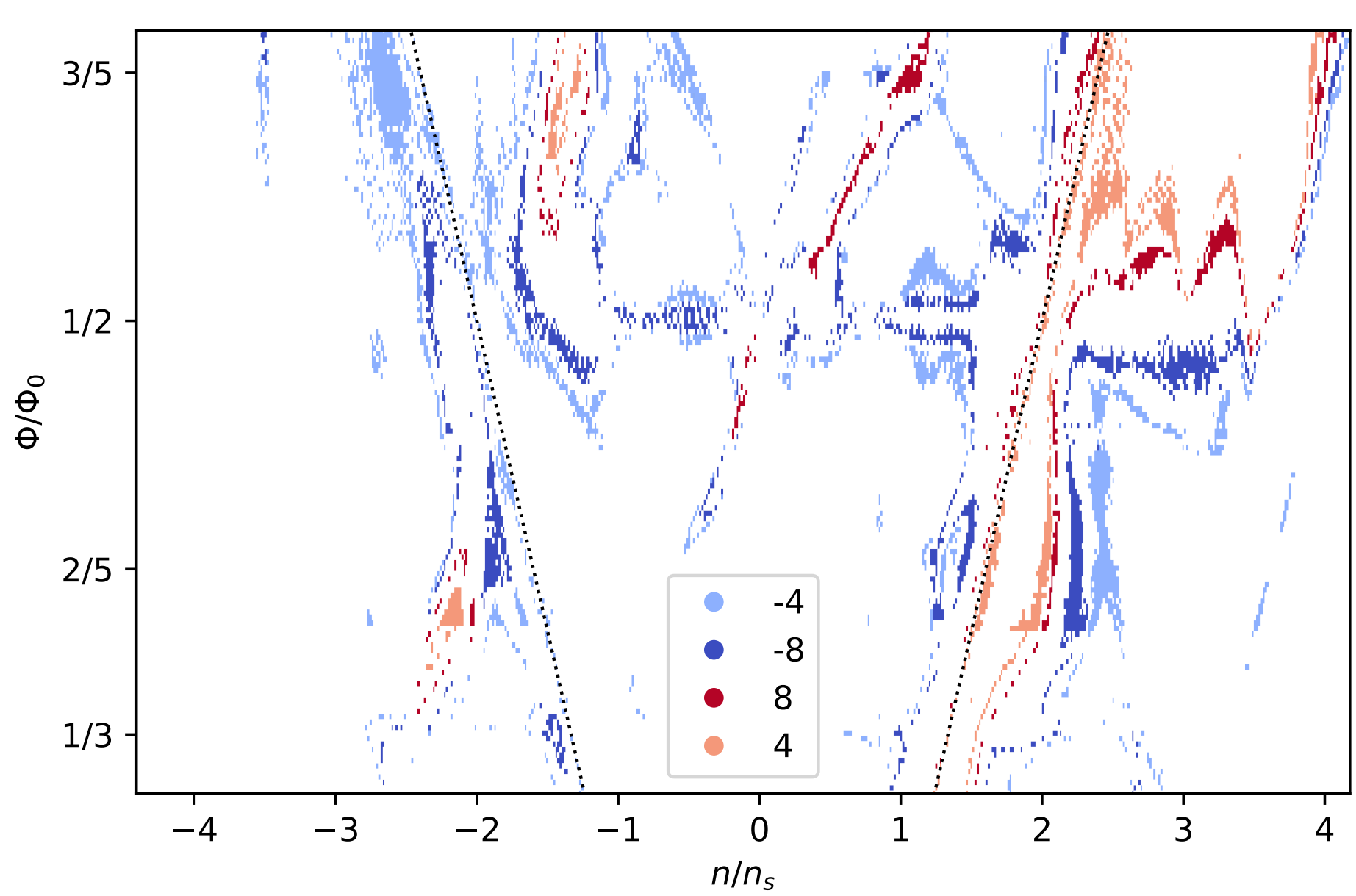

Fig. S16. Integer quantum Hall plateaus. $\pm 200\ \Omega$ windows for several different integer plateaus for contact pair 7 - 17. Dotted lines are (0, pm 4). Though the integer states are reasonably well quantized, they subtend far less of the Landau fan at these fluxes than 8/3.

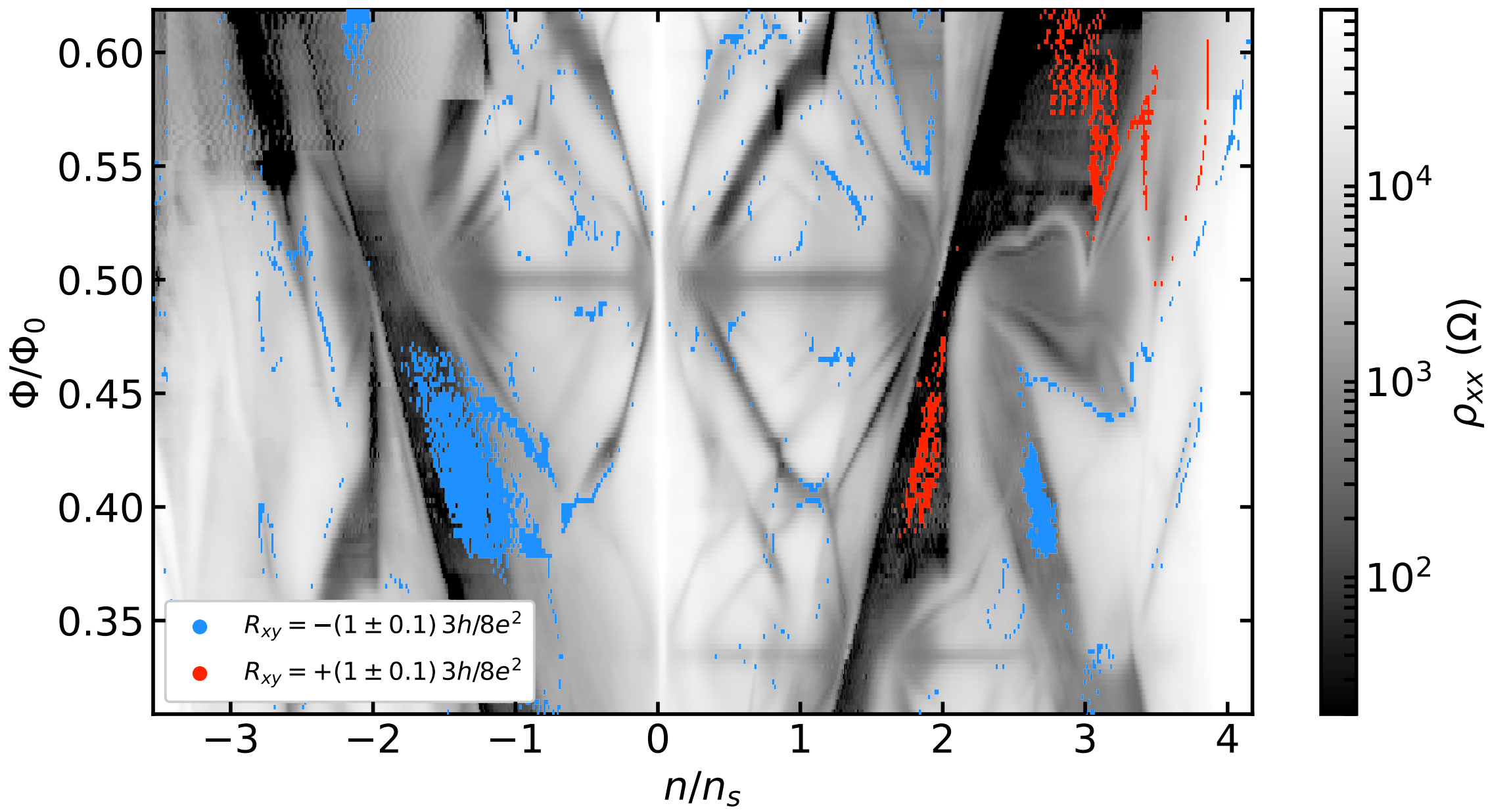

Fig. S17. Raw Hall data. Unfiltered version of Fig. 2.

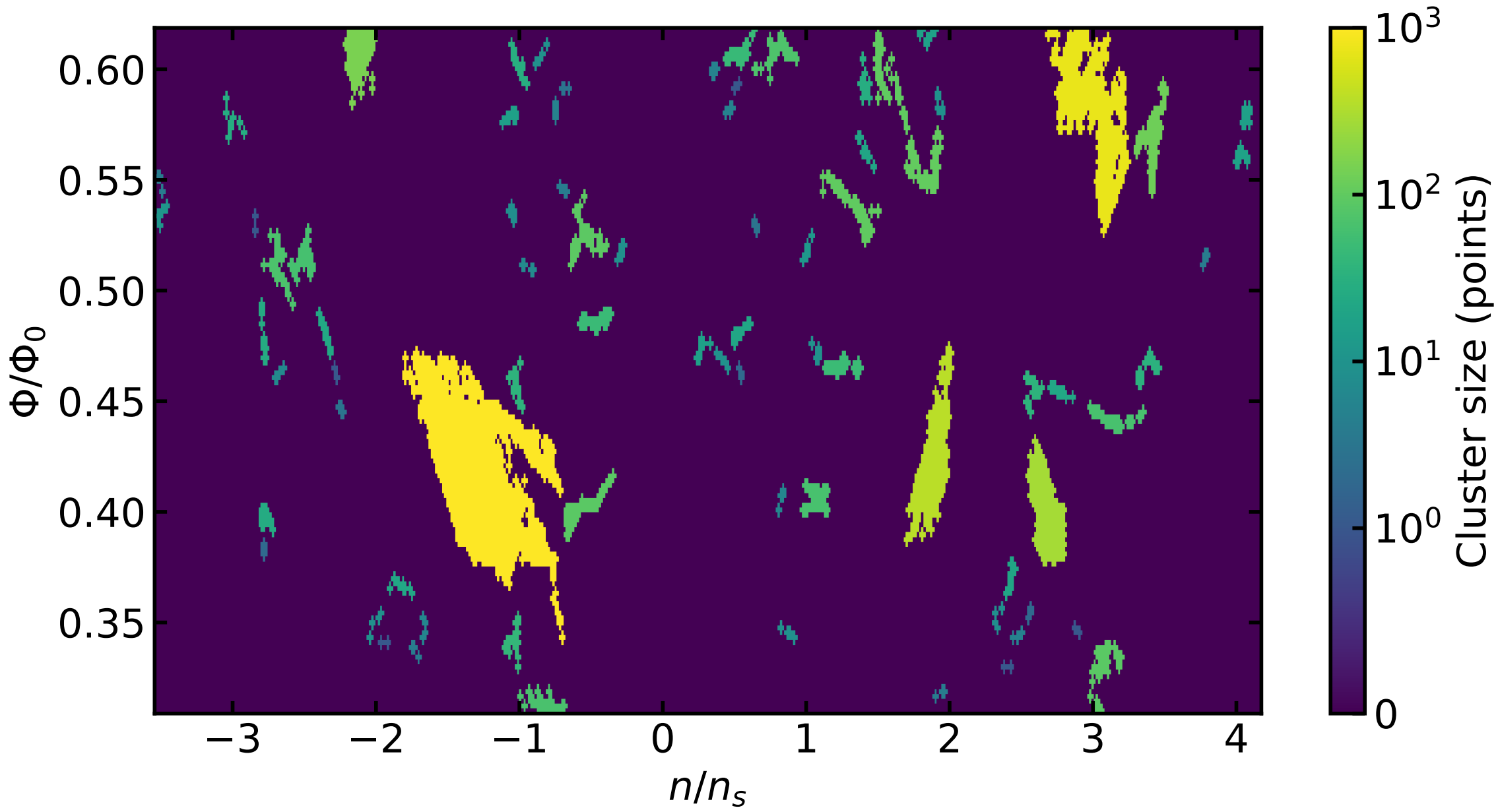

Fig. S18. Clustering mask for $\pm 8/3$. Here we color code clusters by the number of points included in the cluster. Nearest neighbors and next-nearest neighbors that both fall within the defined window will be included in a cluster.

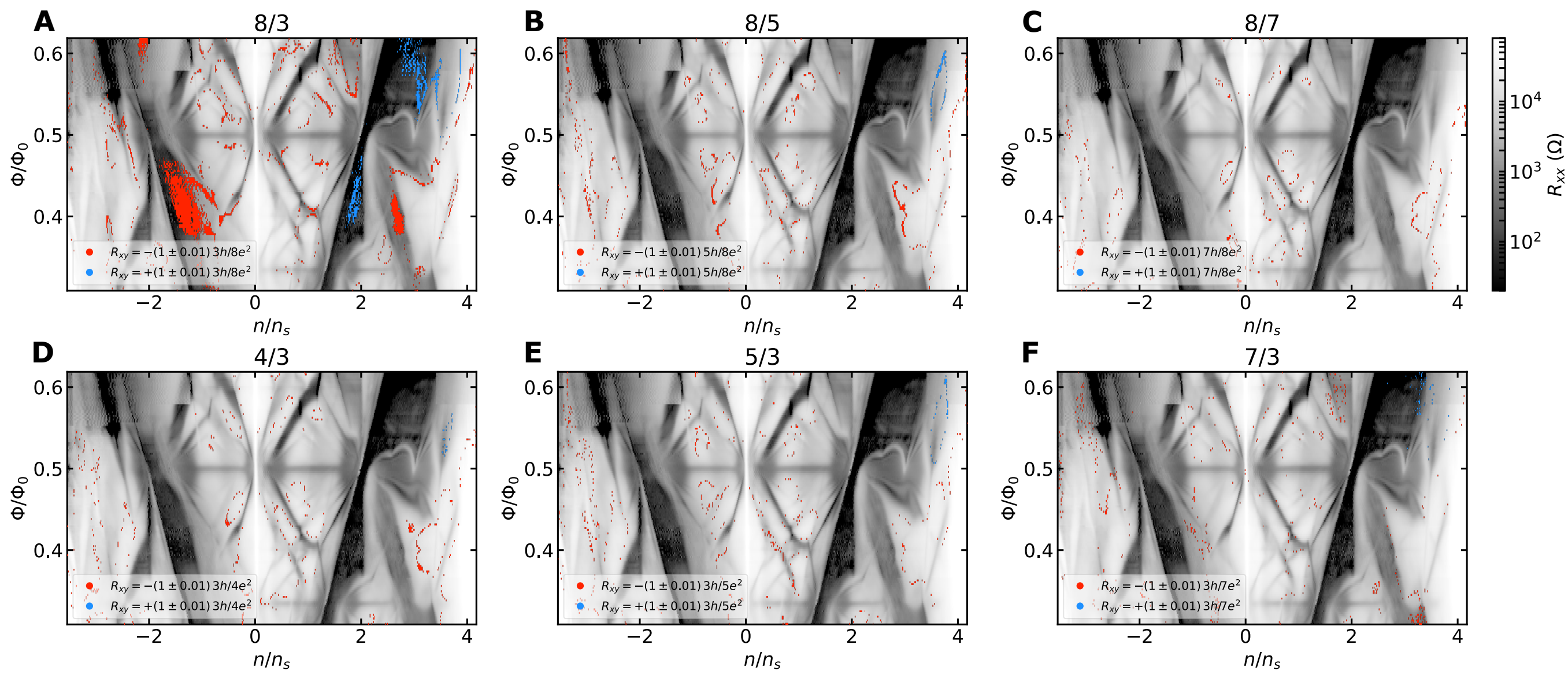

Fig. S19. Highlighting values of Fractional Hall resistance In a similar manner to Fig. 2A of the main text, the longitudinal resistivity is shown in grey-scale. We then highlight regions where the Hall resistance falls within 1% of the specified fractional value: **A** $\pm 8/3$ (reproduced from main text), **B** $\pm 8/5$, **C** $\pm 8/7$, **D** $4/3$, **E** $5/3$, and **F** 7/3. Our clustering algorithm has been applied to remove spurious points (see Sec. S9). Note that the 1% cutoff leads to different sizes of ranges of resistances for each fraction.

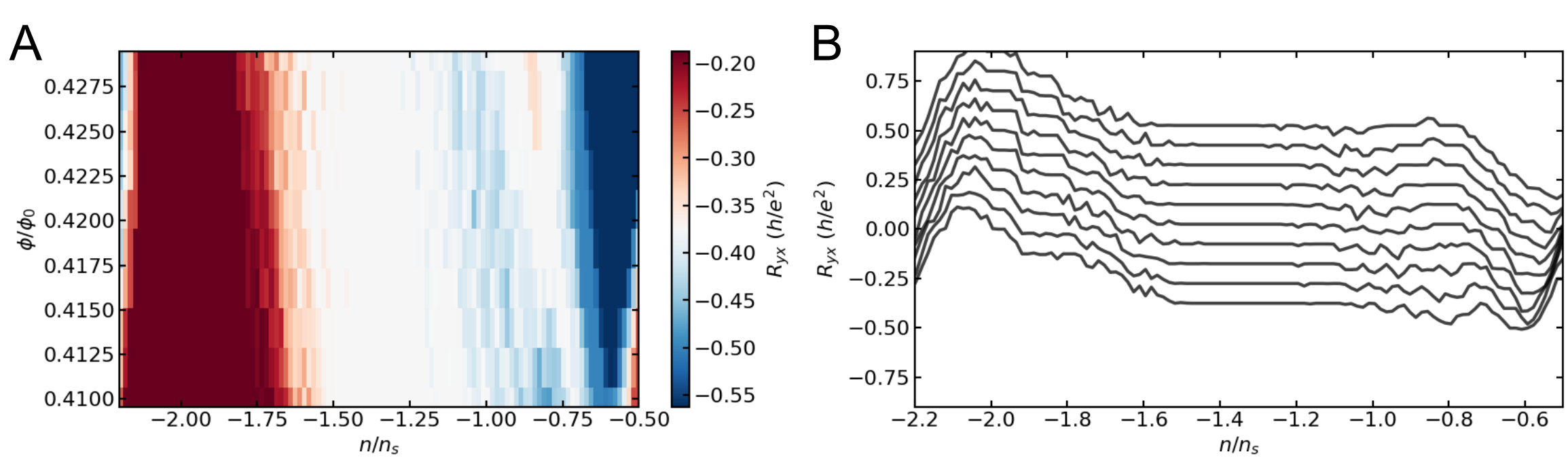

Fig. S20. Possible reentrant FHS. (**A**) Zoomed in colorplot of the Hall resistance in on the largest plateau of -8/3 quantization. Colorbar is centered about $3h/8e^2$, such that white regions are well quantized. (**B**) Waterfall plot of (A) with curves offset for clarity. There is a loss of quantization roughly around -1 n/n_s with quantization recovered on either side.

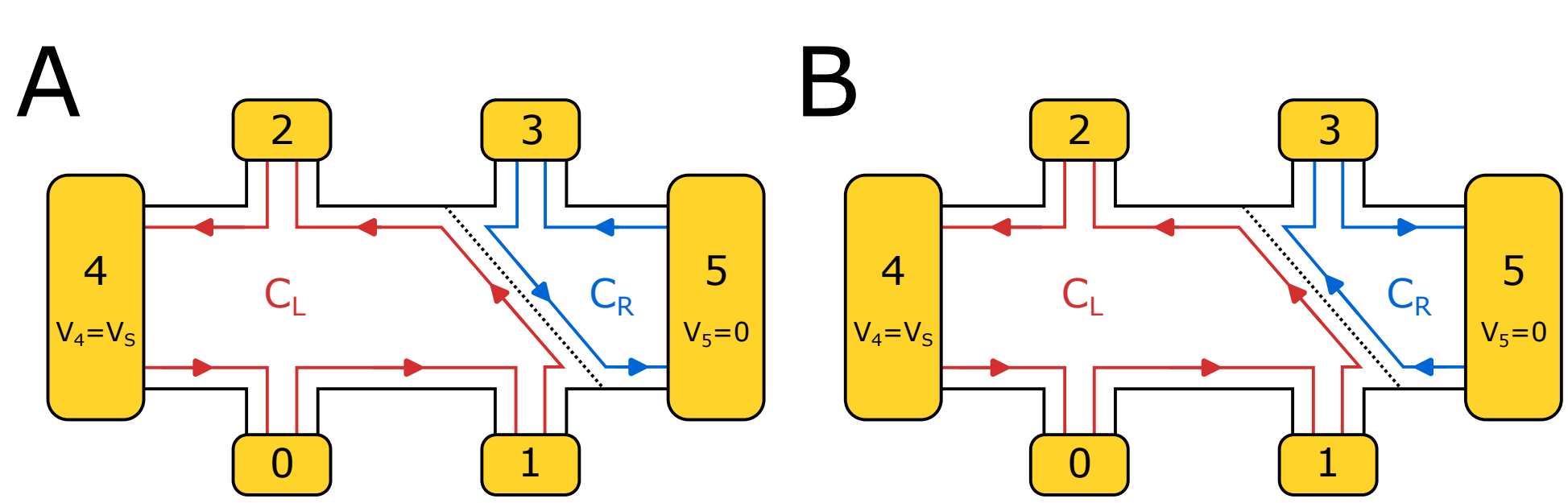

Fig. S21. Hall bar with a domain wall. Schematic diagram of a Hall bar with a domain wall between two Chern insulating regions with different Chern number C_l and C_r for the case of when (**A**) $\mathrm{sgn}(C_l) = \mathrm{sgn}(C_r)$ and (**B**) $\mathrm{sgn}(C_l) \neq \mathrm{sgn}(C_r)$.

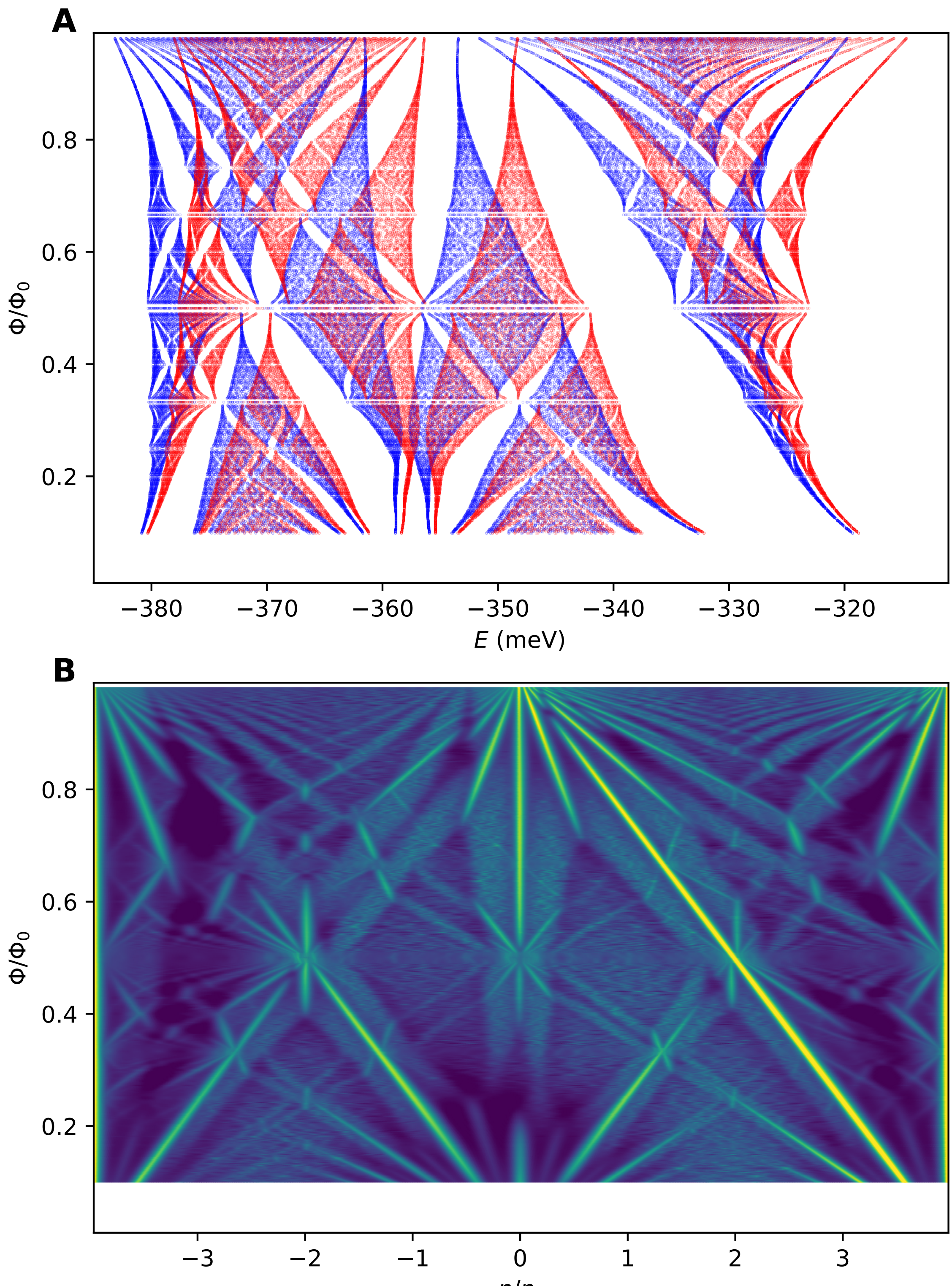

Fig. S22. Hofstadter's butterfly of the strained BM model. (**A**) Computed spectrum for $\theta = 1.38^\circ$, uniaxial heterostrain $\epsilon = 0.2\%$ at 0°angle, and $g = 2$. Red means spin down, blue means spin up. (**B**) Computed inverse density of states.

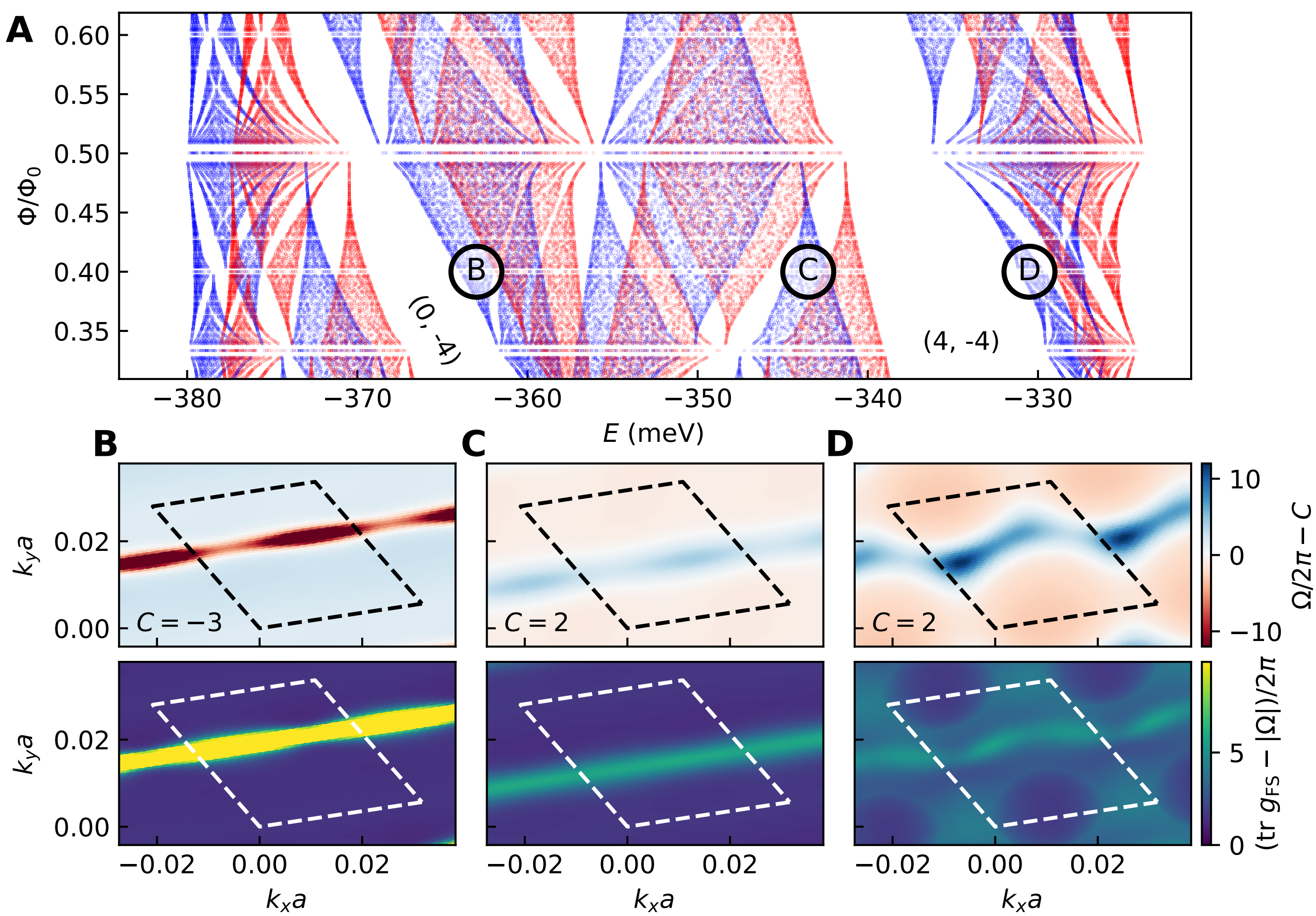

Fig. S23. Computed Hofstadter spectrum and quantum geometric tensor. (**A**) Computed energy levels for $\theta = 1.35°$ with 0.24% uniaxial heterostrain at 45° and 0.3% biaxial heterostrain ($q \leq 72$), as described in the text. Spin up electrons are shown in blue, and spin down electrons are shown in red. The gaps at $(s, t) = (0, -4)$ and $(4, -4)$ are labeled. See Fig. S22 for the full range of fields. (**B**-**D**) Berry curvature (top panels) and trace condition (bottom panels) for the three labeled bands at 2/5 flux in panel A in the first moiré Brillouin zone. These are labelled α, β, and γ in Fig. 3. The black dashed lines correspond to the magnetic BZ. These three bands host the $-8/3$, $+8/3$, and $-8/3$ FHS within the line cuts in Fig. 2, respectively. The trace and Berry curvatures are dimensionless, having been scaled by the area of the magnetic BZ. The units are not scaled to a unit cell area of $\sqrt{3}\pi^2/2 \approx 8.5$.

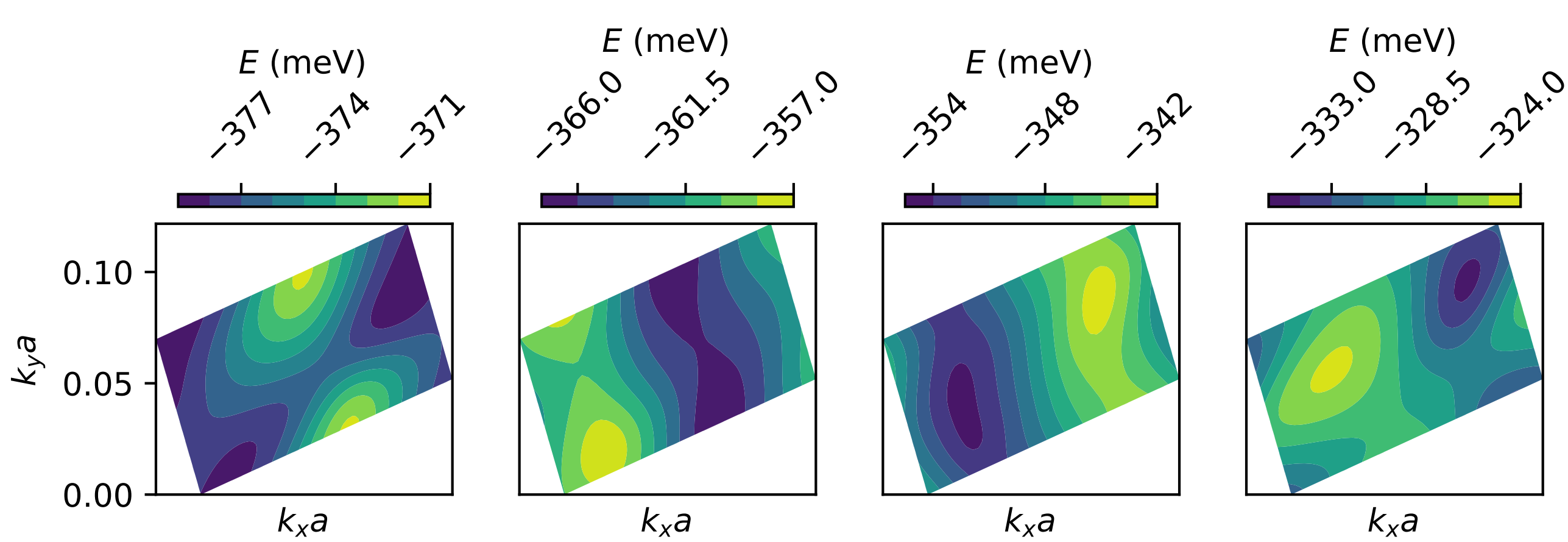

Fig. S24. Computed band structure at 1/2 flux. Computed energy levels for the four bands at half flux. The middlemost bands have regions of open orbits.

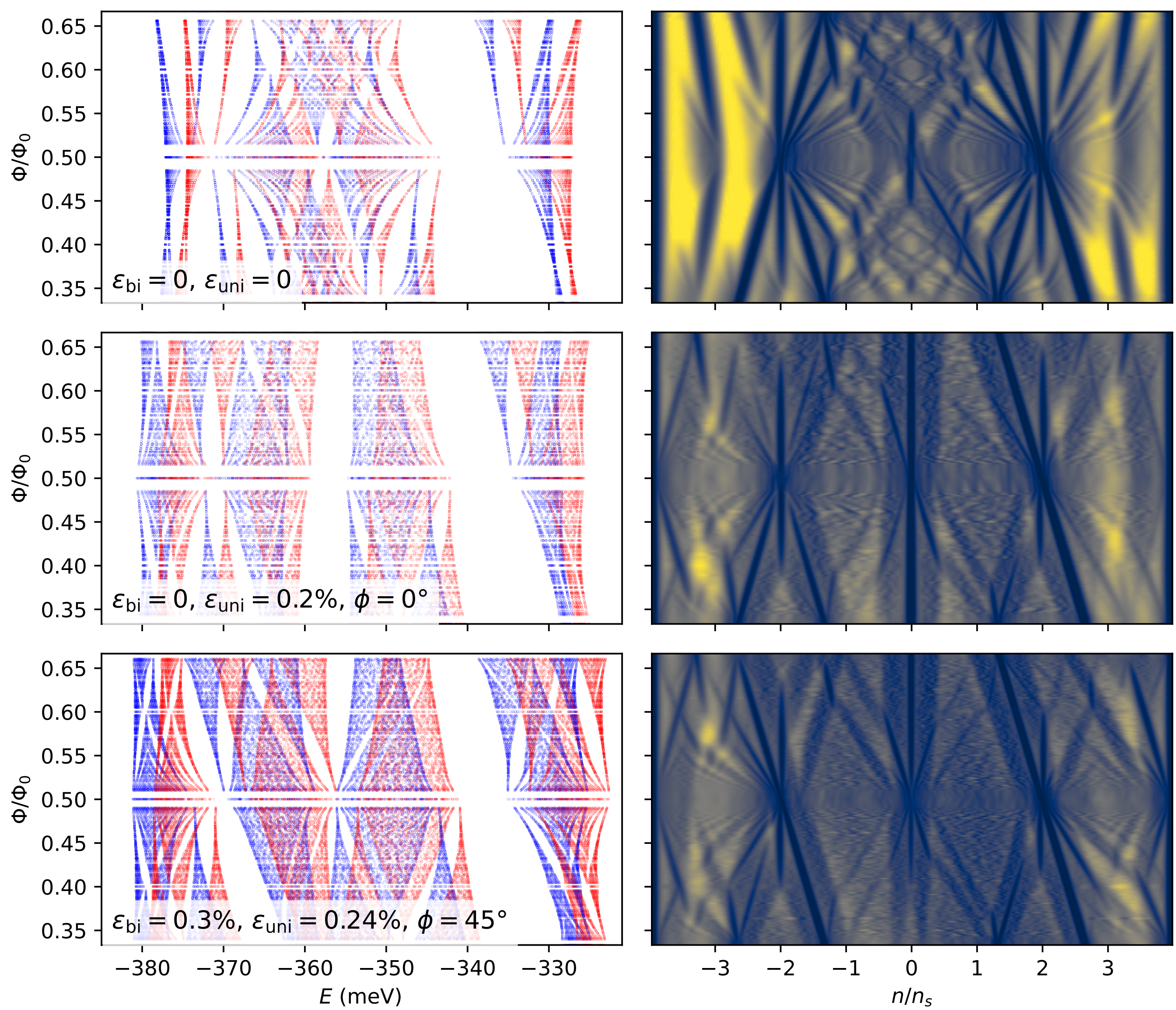

Fig. S25. Computation comparison with different strain matrices. (Left panels) Spectra for the indicated strain parameters. (Right panels) Associated Wannier plots.

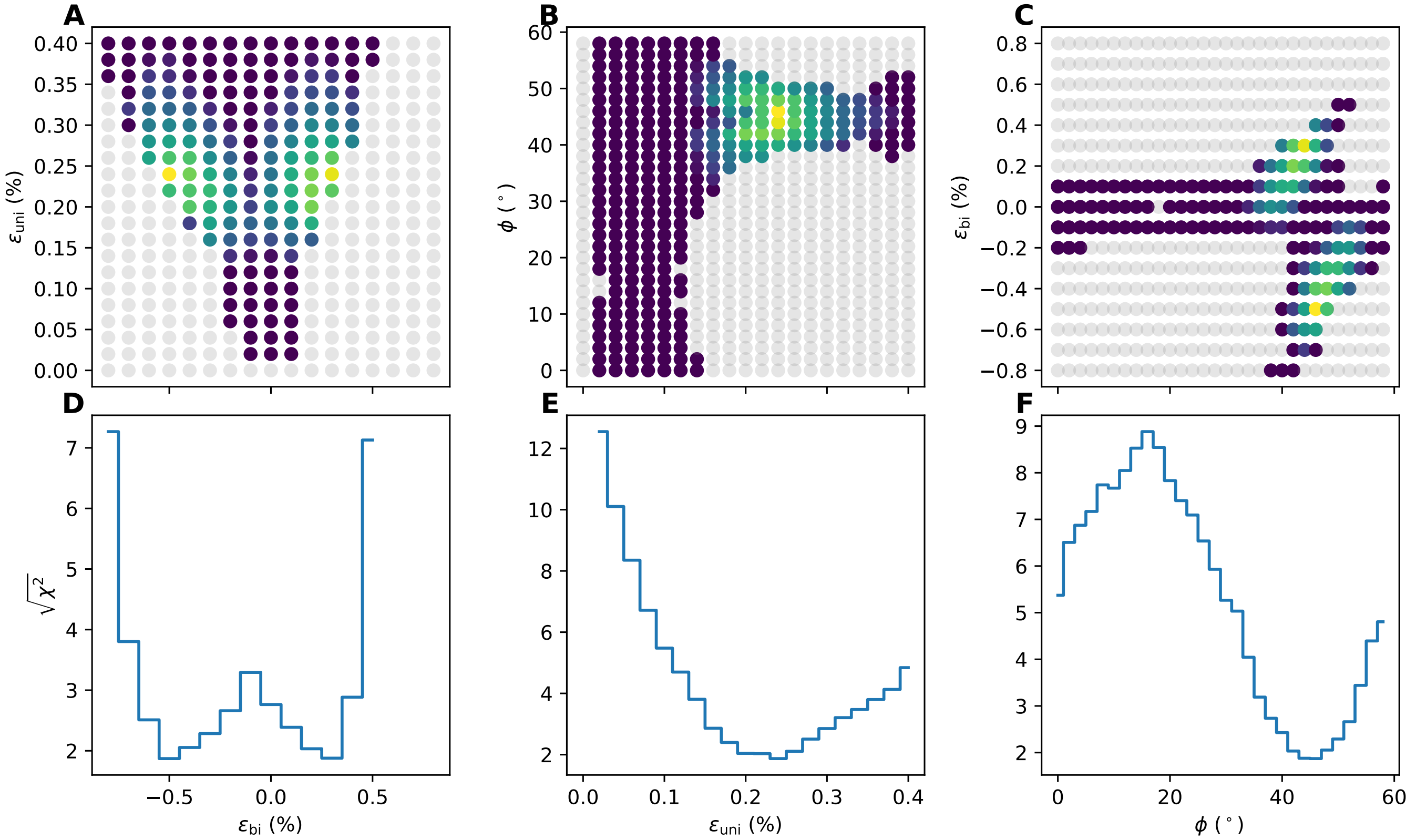

Fig. S26. Best fit to low-field van Hove points. (**A-C**) χ^2 metric for the two indicated parameters (yellow is low, purple is high), using the best value of the not shown third dimension. (**D-F**) One dimensions χ^2 metric, using the best value of both not shown dimensions.

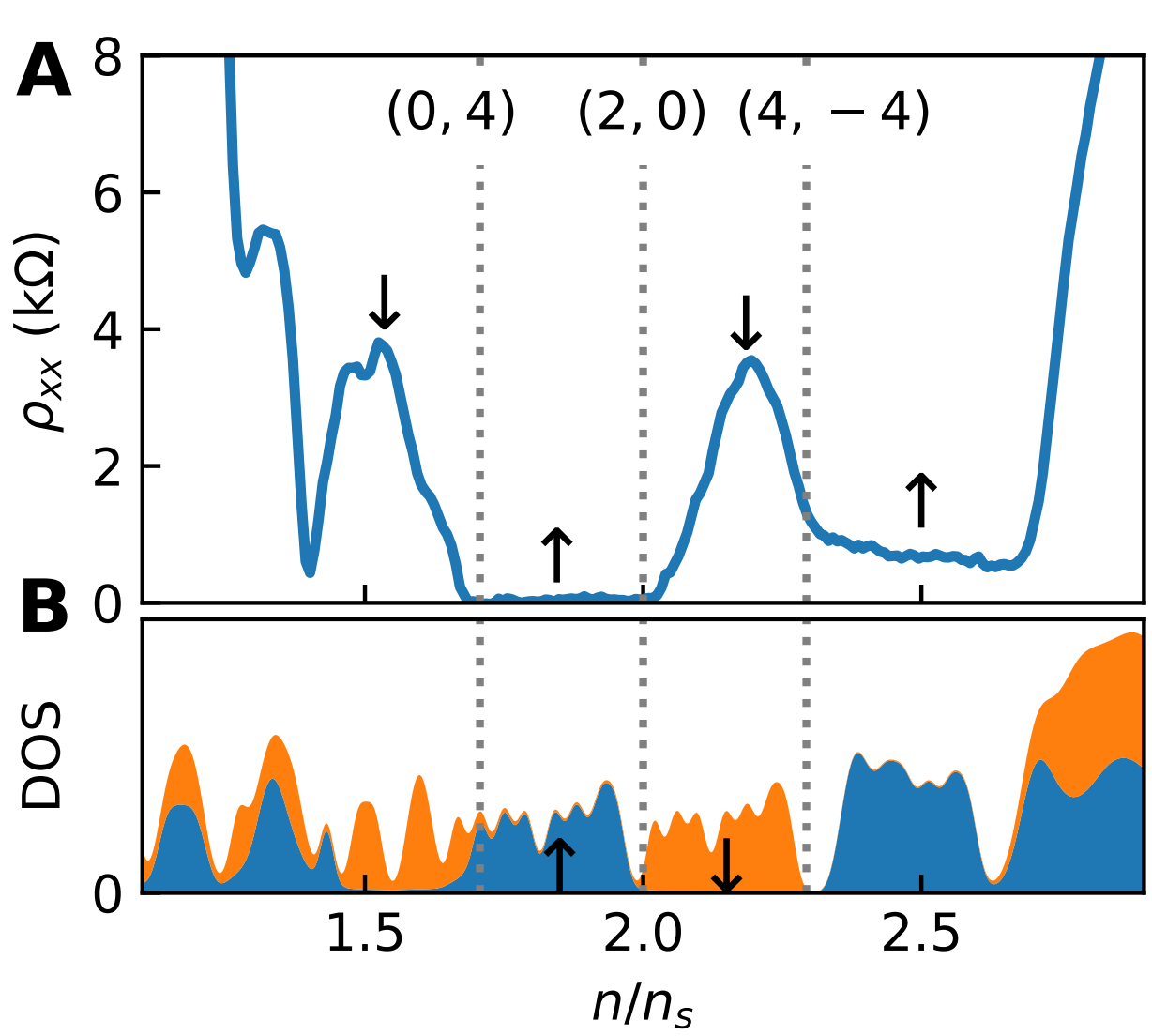

Fig. S27. Possible spin-dependent transport. (**A**) Line cut from Fig. 1 at 19.2 T. Intersections with the $(0,4)$, $(2,0)$, and $(4,-4)$ Hofstadter gaps are indicated with vertical dotted lines. (**B**) Density of states at $p/q = 19/45$ ($B \approx 19.1$ T). Spin up and down states are indicated in blue and orange respectively, as well as with arrows in both panels.

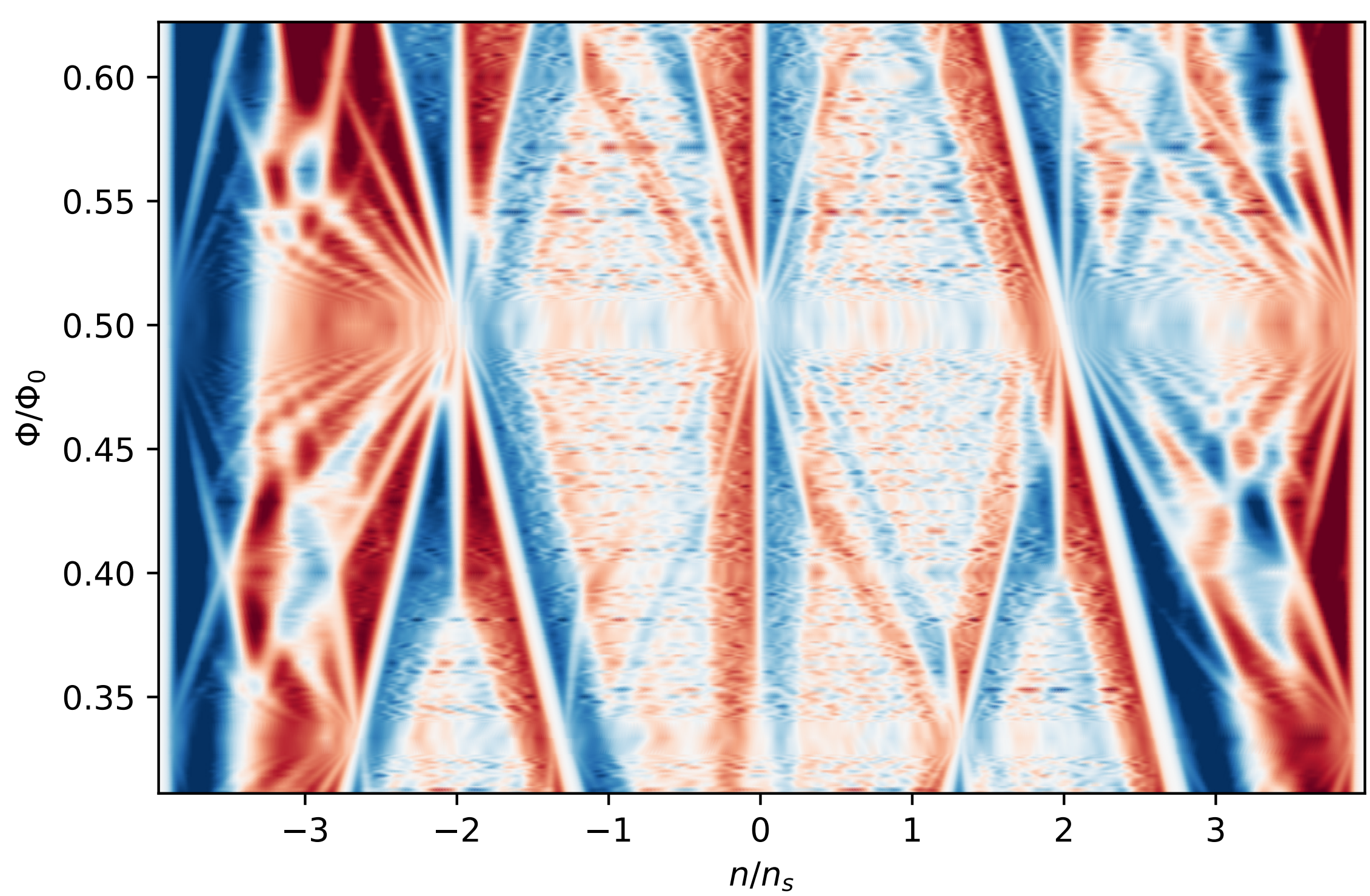

Fig. S28. Spin states in computation. Density of states for spin up minus spin down. Blue means more spin-up than spin down, and red is the opposite.

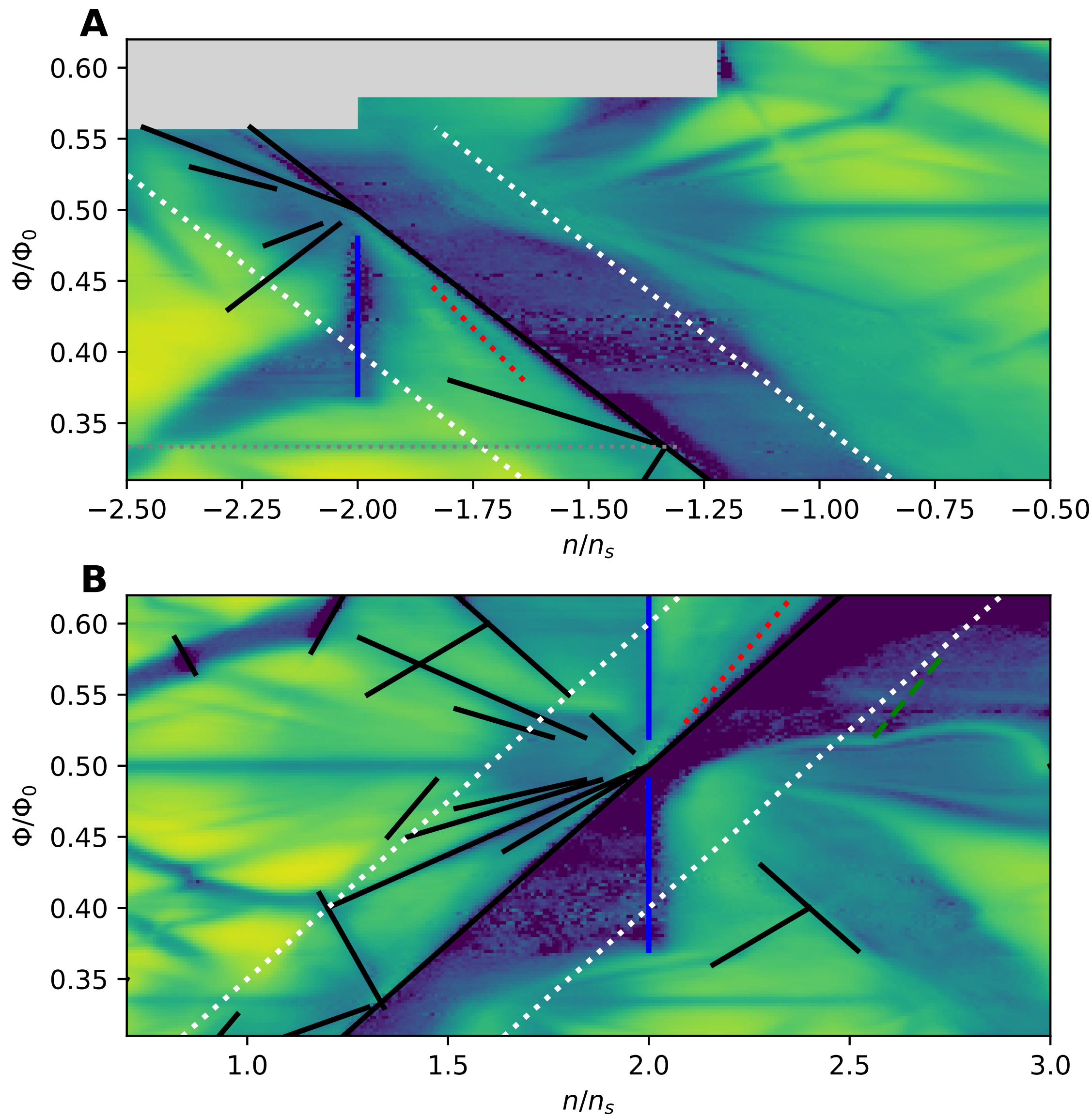

Fig. S29. Spin-dependent transport detail. Zoomed in from Fig. 1. On the left side of $(0, -4)$ (**A**) and $(0, 4)$ (**B**), the resistance is higher than on the right side and there are Středa lines (black lines). Dotted white lines approximate where we start to have two spin species. See Fig. S28.
